# Unlocking Novel Functionality: Pseudocapacitive Sensing in MXene-Based Flexible Supercapacitors

**DOI:** 10.1007/s40820-024-01567-2

**Published:** 2024-12-09

**Authors:** Eunji Kim, Seongbeen Kim, Hyeong Min Jin, Gyungtae Kim, Hwi-Heon Ha, Yunhui Choi, Kyoungha Min, Su-Ho Cho, Hee Han, Chi Won Ahn, Jaewoo Roh, Il-Kwon Oh, Jinwoo Lee, Yonghee Lee

**Affiliations:** 1 Department of Chemical and Biomolecular Engineering, Korea Advanced Institute of Science and Technology (KAIST), 291 Daehak-Ro, Yuseong-Gu, Daejeon, 34141 Republic of Korea; 2 National Nano Fab Center (NNFC), 291 Daehak-Ro, Yuseong-Gu, Daejeon, 34141 Republic of Korea; 3 Department of Organic Materials Engineering, Chungnam National University, 99 Daehak-Ro, Yuseong-Gu, Daejeon, 34134 Republic of Korea; 4 Department of Materials Science and Engineering, Chungnam National University, 99 Daehak-Ro, Yuseong-Gu, Daejeon, 34134 Republic of Korea; 5 Department of Nano & Advanced Materials Science and Engineering, Kyungpook National University, 2559, Gyeongsang-Daero, Sangju-Si, Gyeongsangbuk-Do 37224 Republic of Korea; 6 Department of Mechanical Engineering, Korea Advanced Institute of Science and Technology (KAIST), 291 Daehak-Ro, Yuseong-Gu, Daejeon, 34141 Republic of Korea

**Keywords:** MXenes, Supercapacitors, Pseudocapacitive sensing, Local pH, Operando measurements

## Abstract

Extensively explored for their distinctive pseudocapacitance characteristics, MXenes, a distinguished group of 2D materials, have led to remarkable achievements, particularly in the realm of energy storage devices. This work presents an innovative Pseudocapacitive Sensor. The key lies in switching the energy storage kinetics from pseudocapacitor to electrical double layer capacitor by employing the change of local pH (-log[H^+^]) in MXene-based flexible supercapacitors during bending. Pseudocapacitive sensing is observed in acidic electrolyte but absent in neutral electrolyte. Applied shearing during bending causes liquid-crystalline MXene sheets to increase in their degree of anisotropic alignment. With blocking of H^+^ mobility due to the higher diffusion barrier, local pH increases. The electrochemical energy storage kinetics transits from Faradaic chemical protonation (intercalation) to non-Faradaic physical adsorption. We utilize the phenomenon of capacitance change due to shifting energy storage kinetics for strain sensing purposes. The developed highly sensitive Pseudocapacitive Sensors feature a remarkable gauge factor (GF) of approximately 1200, far surpassing conventional strain sensors (GF: ~ 1 for dielectric-cap sensor). The introduction of the Pseudocapacitive Sensor represents a paradigm shift, expanding the application of pseudocapacitance from being solely confined to energy devices to the realm of multifunctional electronics. This technological leap enriches our understanding of the pseudocapacitance mechanism of MXenes, and will drive innovation in cutting-edge technology areas, including advanced robotics, implantable biomedical devices, and health monitoring systems.

**Supplementary Information:**

The online version contains supplementary material available at 10.1007/s40820-024-01567-2.

## Highlights

- We have discovered a novel phenomenon where the pseudocapacitance of flexible MXene supercapacitors changes sensitively in response to bending, leading to the development of Pseudocapacitive Sensors.
- Pseudocapacitive Sensors repurpose supercapacitors as strain sensors, detecting capacitance changes from shifts between pseudocapacitance and electrical double layer capacitor. These highly sensitive sensors have a gauge factor of about 1200, far exceeding that of conventional strain sensors.

**Supplementary Information:**

The online version contains supplementary material available at 10.1007/s40820-024-01567-2.

## Graphical abstract

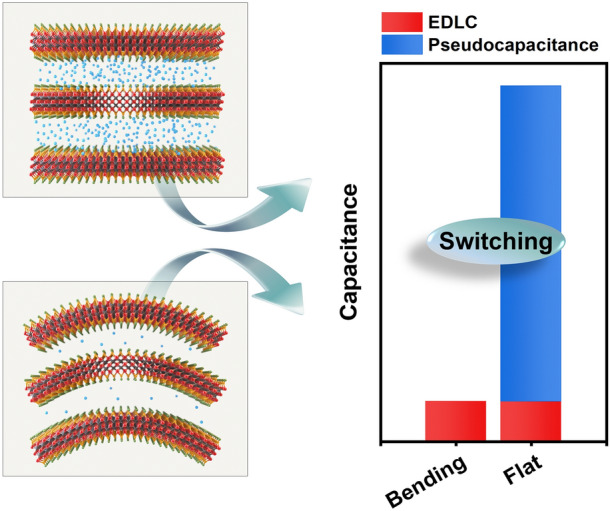

## Introduction

MXenes, owing to their unique pseudocapacitance traits, have been extensively researched in the field of energy storage devices, particularly in supercapacitors and microsupercapacitors (MSCs) [1–7]. Ti_3_C_2_T_x_ MXenes, renowned for their remarkable pseudocapacitive properties, exhibit this phenomenon within supercapacitors due to redox reactions at the electrode–electrolyte interface [8–13]. Specifically, these reactions involve the transfer of charge, resulting in the alteration of the titanium oxidation state, and are accompanied by protonation of oxygen functional groups [14–17]. This phenomenon is compared with that of an electrical double layer capacitor (EDLC), which operates through non-faradaic physisorption without involving any redox chemical reaction.

The potential applications of this distinctive feature extend across diverse fields, specifically in energy storage and power supply systems, eliciting substantial interest. MXene has inherent anisotropic mechanical properties. MXene-hydrogel, containing negatively charged unilamellar nanosheets, aligns under mechanical shearing of Ti_3_C_2_T_x_ liquid–crystal phase.

Meanwhile, in the field of mechanical strain sensor, there are still challenges in practical use for motion sensing as human–machine interfaces: sensitivity, responsivity, hysteresis characteristics (response/recovery), linearity (sensitivity to strain). Among these, the most critical limitation is low sensitivity, where gauge factor (GF) is theoretically 1 [18–20].

Xu et al. improved sensor sensitivity by using ionically cross-linked polymer-alginate and covalently cross-linked polymer-polyacrylamide, achieving a gauge factor (GF) of approximately 165 [21]. Rao et al. fabricated a highly sensitive capacitive strain sensor comprising a self-healing polydiacetylene-polyacrylic acid- Cr^3+^ hydrogel, which exhibits a GF of up to 160 [22]. However, these level of sensitivities are still not adequate for practical applications.

Here, we present a pioneering innovation centered around the creation of Ti_3_C_2_T_x_ MXene-Pseudocapacitive Sensor based on flexible MXene MSCs. The Pseudocapacitive Sensor repurposes ‘supercapacitors’ to function as ‘strain sensors’ by detecting variations in capacitance resulting from the shift in energy storage kinetics during bending. The shift in energy storage kinetics in flexible MXene MSCs is induced by differences in local pH (-log[H^+^]) between flat and bent states. Notably, the presence of the pseudocapacitive sensing attribute distinctly appears in the acidic electrolyte but is absent in the neutral electrolyte. This indicates that the sensing phenomenon correlates with the involvement of H^+^ in the electrochemical reaction, leading to the emergence of pseudocapacitive characteristics.

When shear force is applied during bending, quasi-crystalline 2D MXene nanosheets exhibited structural ordering in flexible MXene MSCs at 2 M PVA/H_2_SO_4_. Due to this, the mobile H^+^ participating in protonation decreases because of the higher diffusion barrier. Consequently, the energy storage kinetics change from pseudocapacitor to EDLC, leading to a decrease in total capacitance. Our in-operando analysis further confirmed the changes in the titanium oxidation state and protonation of oxygen functional groups during bending, providing detailed insights into the underlying electrochemical mechanisms.

In our work, the MXene-Pseudocapacitive Sensor exhibits an ultra-high sensitivity (*S*) of approximately 1200 GF (gauge factor), whereas the conventional dielectric-cap sensor theoretically only achieves 1 GF. This highly sensitive MXene-Pseudocapacitive Sensor offers a new breakthrough to overcome existing limitations in the practical application of strain sensors.

Our research demonstrates the evolution of MXene supercapacitors into multifunctional devices capable of multitasking within a single component, functioning both as energy storage and strain sensors. Enabled by the unique property of pseudocapacitance in MXene, this progress not only innovates upon the limitations of conventional components but also signifies a paradigm shift in the research field, with significant implications for cutting-edge applications including advanced robotics, implantable biomedical devices, and health monitoring systems.

## Experimental Section

### Fabrication

#### Fabrication of Flexible MXene Microsupercapacitors and MXene Pseudocapacitive Sensors

Ti_3_C_2_T_x_ MXene solution was prepared at a concentration of 15 mg mL^−1^. The synthesis method used the following LiF/HCl MILD method [22].

Polyimide (PI) film was laminated onto a Si-wafer as a flexible substrate. A negative PR mask, specifically L300, was prepared on the PI film through UV exposure and development. The interdigitated pattern specifications include a finger width of 50 μm, inter-electrode spacing of 50 μm, finger length of 3.95 mm, thickness of 3.5 μm, and a total of 60 fingers. Subsequently, O_2_ plasma treatment was applied to the PR mask to enhance hydrophilicity. Next, as-prepared 15 mg mL^−1^ of MXene solution was spin-coated at 1000–1500 rpm for 5 min. The lift-off process was executed by immersing the sample in acetone and subjecting it to sonication to remove the PR and achieve the interdigitated MXene pattern. A 10 wt% polyvinyl alcohol (PVA) gel was then spin-coated at 1000 rpm for 5 min as a buffer layer on the interdigitated MXene pattern. The resulting MXene flexible microsupercapacitors (FMSCs) were completed by dropping PVA/H_2_SO_4_ gel electrolyte. The molar concentration of the PVA/H_2_SO_4_ gel electrolyte was controlled in a range from 0.5 to 4 M. After application, the MXene FMSC is allowed to dry for about 6 h to ensure complete adhesion and stability.

#### Fabrication of Flexible MXene Microsupercapacitors

After activating the as-fabricated MXene FMSC through the infiltration of H^+^ ions between MXene sheets, it was then applied as an MXene-Pseudocapacitive Sensor. The activation process was performed by repetitive charging and discharging when bending and releasing several times. MXene-EDLC underwent the same process.

### Characterization

#### Conventional Method

To nano-characterize Ti_3_C_2_T_x_ MXene nanomaterial, transmission electron microscopy (TEM, Tecnai G2 F30 S-TWIN, FEI), Zeta potential/DLS (Zetasizer nano zs, Malvern), X-ray diffraction (XRD, SmartLab, Rigaku corporation), X-ray photoelectron spectroscopy (XPS, Nexsa G2, Thermofisher Scientific), and Raman spectroscopy (NS200, Nanoscope systems) were utilized as structural and chemical intrinsic properties. The configuration of MXene MSCs was measured by scanning electron microscopy (SEM) and energy dispersive spectroscopy (EDS) (SU8230, Hitachi High-Technologies Corp.) and atomic force microscopy (AFM, XE-100, Park Systems).

#### Ex-Situ Method

To compare the changes of Ti_3_C_2_T_x_ MXene as structural and chemical after charge/discharge, XRD and XPS were employed, respectively. The change in *d*-spacing was investigated by XRD, while the distribution of the functional group was performed through XPS. Additionally, the change in MXene alignment in flat and bent states was analyzed by grazing-incidence wide-angle X-ray scattering (GIWAXS, NANOPIX, Rigaku).

#### In-Situ Operando Method

The XPS sampling of flexible MXene/PVA-H_2_SO_4_/MXene was conducted on a flexible PET substrate, featuring an exposed hole on the MXene surface. For the analysis, a micro-focused monochromatic Al Kα X-ray source (1486.7 eV) was utilized alongside a spherical sector analyzer and 3 multichannel resistive plate detectors with 128 channels. The setup aimed to minimize data acquisition time for real-time connection to the power supply.

XPS data acquisition focused on Ti 2*p*, O 1*s*, and C 1*s* from the top surface of the flexible MXene/PVA-H_2_SO_4_/MXene symmetric cells under ultrahigh vacuum (UHV) conditions. The electron take-off angle was set at 90°, while the X-ray incident angle was 30° relative to the sample surface. The XPS data recording was carried out at 75 W with an X-ray beam size of 400 × 200 µm^2^. Potentials were applied using a potentiostat/galvanostat/impedance analyzer (ZIVE SP1, WonATech).

The collection conditions involved maintaining a base pressure of 1.0E-09 mbar, a voltage range of 0 – 0.6 V for MXene/PVA-H_2_SO_4_/MXene symmetric-cells, and snapshot mode at a pass energy of 150 eV. To ensure electronic charge neutralization, a dual-beam low-energy electron–ion source was employed.

### Electrochemical Measurement

The electrochemical measurements (i.e., CV, GCD) were conducted using a Biologic VSP parameter analyzer. The CVs were conducted at various scan rates and the GCDs were carried out at different current densities.

The volumetric specific capacitance was calculated by the CV curves using the following equation [6, 23]:

1 $$C = \frac{1}{{\nu \times \left( {V_{f} - V_{i} } \right) \times V}} \mathop \int \limits_{{V_{i} }}^{{V_{f} }} I\left( V \right)dV$$

where $\nu$ is the scan rate, ${V}_{f}$ and ${V}_{i}$ are the integration potential limits of the CV curve, and $I\left(V\right)$ is the discharging current. *V* is the effective volume of the electrode.

The specific capacitance was calculated by the GCD curves as follows:

2 $$C = \frac{4I}{{dV/dt \times V}}$$

where $I$ is the applied current for discharging test and $dV/dt$ is the slope from the discharge curve (excepting the IR drop region). *V* is the effective volume of the electrode.

### Mechanical Sensing Test

The sensing tests were conducted using a bending machine (bending & stretching tester, HANKOOKLAB) connected to an LCR meter (IM3536 LCR meter, HIOKI). The sensing test was carried out by repetitive bending and releasing with each cycle lasting 10 s. The capacitance value measured by the LCR meter was recorded in real-time. LCR meter was utilized with a DC bias of 2 V and a frequency range of 4 Hz to 4 MHz.

## Results and Discussion

**Scheme 1:**
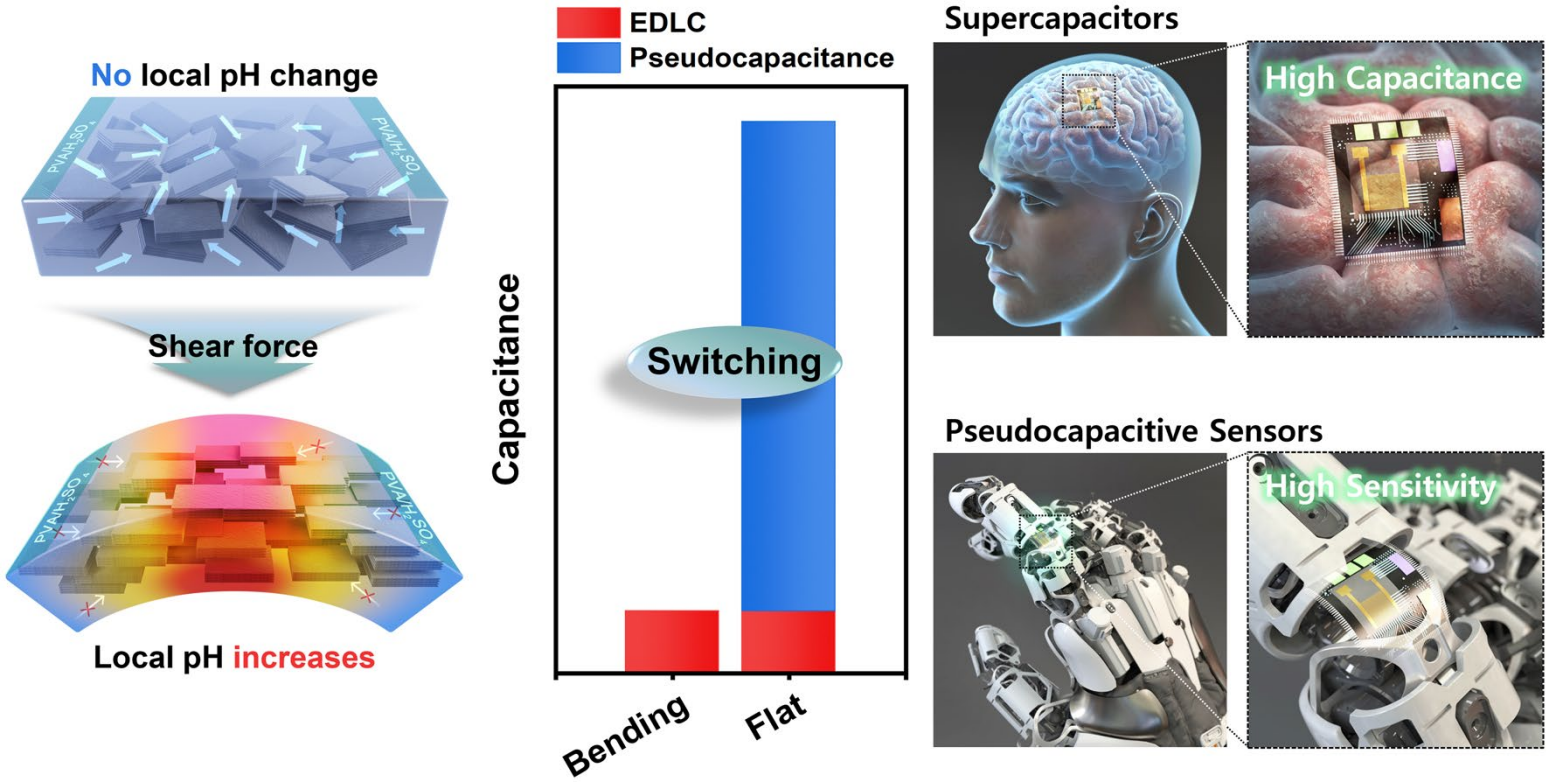
Schematic illustrations of flexible MXene-based Pseudocapacitive Sensors, demonstrating the changes in pseudocapacitance/EDLC (electric double-layer capacitance) of MXene supercapacitors when bent and released

In Scheme 1, we demonstrate flexible MXene-based multifunctional electronics, which serve dual roles: functioning as supercapacitors for micro-energy applications and as Pseudocapacitive Sensors for micro-sensor applications. Pseudocapacitive sensing, as defined in this work, is characterized by altering the charge storage kinetics through diffusion intercalation and capacitive adsorption, influenced by the distribution of H^+^ ions. In the released (flat) state, a high concentration of H^+^ ions facilitates the device's role as MSCs; however, when in the bending state, a reduced distribution of H^+^ ions prompts its function as a Pseudocapacitive Sensor, which is detailed further in subsequent sections.

### Characterization of Ti_3_C_2_T_x_ MXene and Configuration of MXene-Based Flexible MSCs

**Fig. 1:**
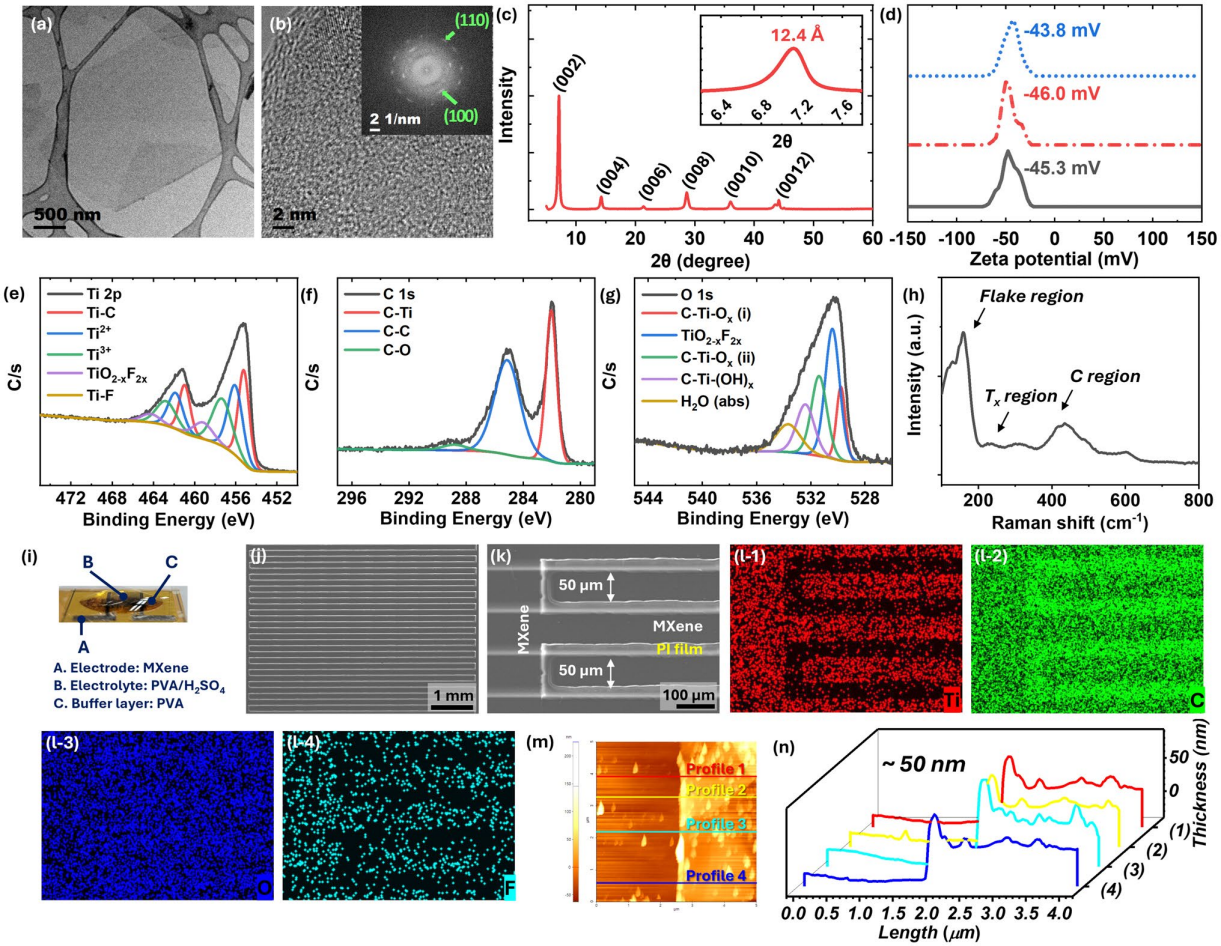
TEM images of MXene nanosheets at **a** low magnification and **b** high magnification (inset of FFT pattern). **c** XRD patterns ranging from 3° to 60° (inset: narrow range from 6.2° to 7.8°) and **d** zeta potential for MXene solution. XPS spectra in the **e** Ti 2*p*, **f** C 1*s*, and **g** O 1*s*, and **h** Raman spectra for MXene film. **i** Schematic illustration of MXene-based flexible MSCs. SEM images at **j** low magnification and **k** high magnification, and **l** EDS mapping images of Ti (red color), C (green color), O (blue color), and F (cyan color) elements. **m** AFM image and **n** line profiling (*t* =  ~ 50 nm)

The data presented in Fig. 1a reveals that the flake size of MXene nanosheet is approximately 1 µm with corresponding crystal properties illustrated in the HR-TEM and fast Fourier transform (FFT) patterns displayed in Fig. 1b. XRD analysis confirms the delaminated 2D MXene characteristics, with a measured *d*-spacing value of 12.4 Å at (002) peak of 7.1° (Fig. 1c). The averaged zeta potential (*ζ*) is -45.03 mV (Fig. 1d), indicating an abundance of negatively charged functional groups (T_x_ = -O, -OH, -F) within the MXene layers, crucially formed during the acid etching synthesis process of MAX (Ti_3_AlC_2_) to MXene (Ti_3_C_2_T_x_).

The XPS spectra of Ti_3_C_2_T_x_ in Fig. 1e–g shows Ti 2*p* peaks split into Ti 2*p*_3/2_ and Ti 2*p*_1/2_ at approximately 455 and 463 eV, respectively. The Ti 2*p*_3/2_ spectra are fitted into four components corresponding to C-Ti, C-Ti^2+^-(O/OH/F), C-Ti^3+^-(O/OH/F), and TiO_2-x_F_2x_, based on the oxidation states of Ti, at 455.13 (460.88 eV), 456.08 (461.93 eV), 457.33 (462.83 eV), and 459.23 (464.43 eV) [18–20], respectively (Fig. 1e). The first peak was assigned to Ti bonded to C only, while the second and third peaks were assigned to Ti atoms bonded to -O/-OH/-F terminations. The last peak was attributed to TiO_2- x_F_2x_, formed due to the degradation/oxidation of Ti_3_C_2_T_x_ by reaction with ambient air and/or water. Typically, the binding energy of C–C bonds originating from adventitious carbon is at 284.8 eV, irrespective of the type of terminations. The peak at 281.9 eV arises from carbon atoms residing in the Ti octahedral sites (Ti-C-Ti) of Ti_3_C_2_ MXene (Fig. 1f). Figure 1g shows an analysis of the O 1*s* spectra, where the -O terminations at bridge sites (C-Ti-O_x_(i)) and A/B sites (C-Ti-O_x_(ii)) were observed at 529.78 and 531.33 eV, respectively. The -OH termination at A/B sites (C-Ti-(OH)_x_) appeared at 532.43 eV, and two other peaks assigned to TiO_2-x_F_2x_ for organic contamination and absorbed H_2_O were detected at 530.38 and 533.68 eV, respectively. The fitting of the O 1*s* spectra is more complex than that of other elements due to the complexity of -O and -OH terminations in determining their location (A/B site or bridging site) and bonding nature (oxide, hydroxide, oxyfluoride) with the exposed Ti sites [24–26]. Raman spectroscopy of Ti_3_C_2_T_x_ is presented in Fig. 1h, providing insights into the bonding structures and vibrational properties of MXenes. In the fingerprint region spanning 100 – 800 cm^−1^, the spectrum is divided into three regions: the flake, the T_x_, and the carbon [27–29].

Figure 1i shows a digital photograph of MXene-based flexible MSCs. Electrode (A) is MXene which serves as active material and current collector due to these conducting properties. Electrolyte (B) is gel-based acidic PVA/H_2_SO_4_. The buffer layer (C) is PVA which is introduced for enhancing mechanical stability. The micropatterned MXene electrode was fabricated by PR photomasking directly on PI film and MXene spin coating and lift-off. Flexible MXene MSCs were manufactured by loading gel-based PVA/H_2_SO_4_ electrolyte onto MXene micropatterns. Further details about the fabrication process of MXene-based flexible MSCs is provided in the supporting information (Fig. S1).

Figure 1j, k shows SEM images of interdigitated MXene micropatterns on PI film as a flexible substrate. As demonstrated in previous research, the interdigitated planar MXene micropatterns have a length of approximately 4 mm with a finger width of ~ 50 μm and inter-electrode gap of ~ 50 μm [23]. The active area of 30-finger in-plane flexible MXene-based MSCs was around ~ 0.1185 cm^2^. As observed in the EDS color mapping shown in Fig. 1(l-1)-(l-4), the element of Ti (red color) and F (cyan color) is bright at the MXene electrode region following the MXene micropattern, whereas the element of C (green color) is bright at the inter-electrode gap region which is a polyimide film substrate. Figure 1m displays an AFM image focused between the MXene electrode and inter-electrode gap in flexible interdigitated MXene micropatterns. The thickness of the interdigitated MXene electrode, approximately 50 nm, was determined via line profiling through the AFM image, as presented in Fig. 1n.

### Electrochemically Pseudocapacitive Sensing Phenomena

**Fig. 2:**
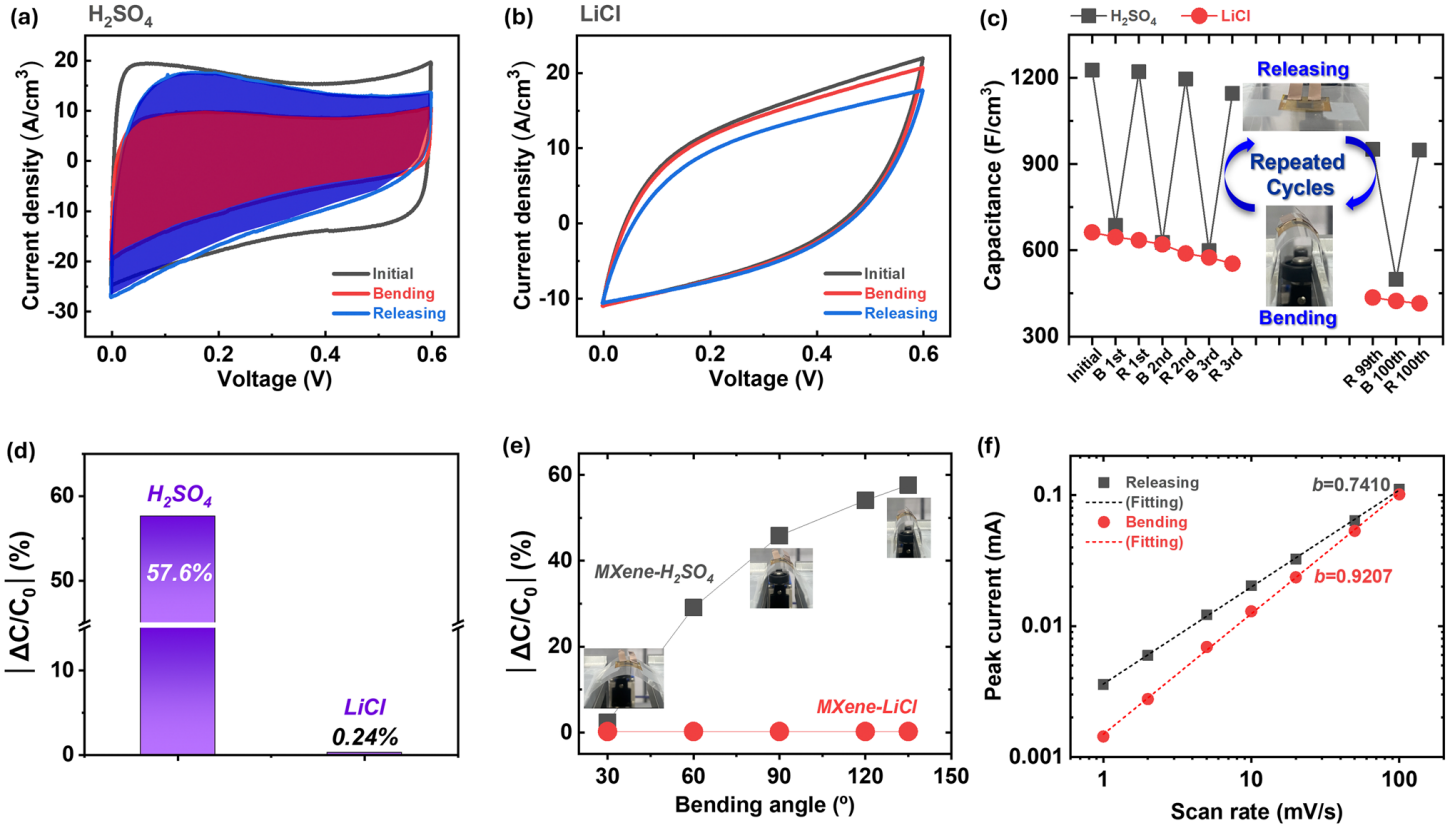
Electrochemical pseudocapacitive sensing phenomenon during bending and releasing. Cyclic voltammetry (CV) curves on **a** 2 M H_2_SO_4_/PVA with pseudocapacitive sensing, and **b** 2 M LiCl/PVA without pseudocapacitive sensing during bending/releasing. **c** Change in capacitance during bending and releasing for up to 100 cycles on different electrolytes, and **d** relative change in capacitance depending on different electrolytes at a bending angle of 90°. **e** Relative change in capacitance on different electrolytes as bending angles differ between 30°–135°. **f** *b*-values as a function of power-law dependence on the current at scan rate

In Fig. 2a, cyclic voltammetry (CV) curves of flexible MXene MSC in acidic PVA/H_2_SO_4_ electrolyte are presented at initial state, bending state, and releasing state at scan rate of 50 mV s^−1^. The rectangular areas in the CV curves represent the capacitance [23], with the red-colored area corresponding to the bending state and the blue-colored area corresponding to the releasing state. It can be observed that the area, *i.e.*, capacitance, on the CV curve decreases when the device is bent. This unique sensing phenomenon is exclusively observed in flexible MXene MSCs operating in acidic PVA/H_2_SO_4_ electrolyte.

In contrast, as shown in Fig. 2b, there was no change in the area, indicating that the capacitance of flexible MXene MSC remained constant in neutral PVA/LiCl electrolyte at scan rate of 50 mV s^−1^. The sensing phenomena are completely absent when using neutral PVA/LiCl, which is a representative electrolyte electrochemically exhibiting electrical double layer capacitance (EDLC) behavior [30, 31]. Similarly, in the galvanostatic charge–discharge (GCD) curves (Fig. S2), the trend parallels that of CV, with no observable change during bending and releasing in PVA/LiCl electrolyte. However, in PVA/H_2_SO_4_ electrolyte, there is a recovery of capacitance from bending to releasing, highlighting the unique sensing behavior observed exclusively in the electrolyte containing H^+^ ions.

Figure 2c shows the capacitance change calculated by CV curves (Fig. 2a, b) of flexible MXene MSCs in PVA/H_2_SO_4_ and PVA/LiCl, respectively. In the PVA/H_2_SO_4_ electrolyte, the capacitance of flexible MXene MSCs exhibits a repetitive pattern of decrease and increase during bending and releasing, with continuous recovery of capacitance. The initial capacitance is 1226.8 F cm^−3^. After activation by infiltrating H^+^ ions, the capacitance decreases to 686.9 F cm^−3^ when bent, then increases to 1221.4 F cm^−3^ upon releasing. Subsequently, the capacitance decreases to 627.2 F cm^−3^ upon re-bending and increases to 1196.0 F cm^−3^ upon re-releasing. In the next cycle, the capacitance decreases and increases from 598.7 to 1145.8 F cm^−3^ upon re-bending and re-releasing, respectively. This demonstrates continuous recovery of capacitance during the bending-to-releasing transition with different CV curve areas.

Conversely, in PVA/LiCl electrolyte, there is no variation in capacitance during bending and releasing, and it gradually deteriorates from 662.5 F cm^−3^ initially to 553.7 F cm^−3^. Relative capacitance change (|ΔC/C_0_|) is 57.6% in PVA/H_2_SO_4_, whereas it exhibits 0.24% in LiCl, indicating a negligible sensing effect in the latter (Fig. 2d). Furthermore, this sensing phenomena occurs regardless of presence or absence of a buffer layer. Figure S3 illustrates the change in capacitance of flexible MXene MSCs without a buffer layer during bending and releasing. The initial capacitance is 946.3 F cm^−3^, decreasing to 291.2 F cm^−3^ upon bending and then increasing to 580.4 F cm^−3^ without fully recovering its initial capacitance.

To investigate the linearity between relative capacitance change and bending angles, indicative of the degree of strain, the capacitance of flexible MXene MSCs was measured across different electrolytes (Fig. 2e). The relative capacitance change in PVA/H_2_SO_4_ varies from 2.39% to 57.61% at bending angles ranging from 30° to 135°, indicating a near linear relationship between bending angles and capacitance change. The relative capacitance change in PVA/LiCl remains constant at 0.236%, irrespective of variations in the bending angles, which range from 30° to 135°.

Regarding the direction of the strain curvature, at a positive curvature (κ =  + 0.263 mm^−1^), the relative capacitance change (│ΔC/C_0_│) is 57.6%, whereas it is 9.5% at a negative curvature (κ = -0.263 mm^−1^) (Fig. S4). Only pseudocapacitive sensing is effective primarily in a positive curvature, corresponding to tensile strain (outward bending), while it is less effective in a negative curvature, corresponding to compressive strain (inward bending). This trend arises from the alteration in the ordering of the *c*-axis alignment of MXene nanosheets under different curvatures, specifically the strain applied to MXene nanosheets.

To further interpret the shift in charge storage kinetics during bending and releasing attributed to differences in proton distribution, we employ a power-law dependence [14, 15, 32, 33]. The total charge stored can be divided into two main contributions: the (i) surface capacitive process and (ii) diffusion-limited Faradaic intercalation process. Supposing the power-law dependence of the current *I* on scan rate *v* is measured from CVs [14, 15, 32, 33]:

3 $$i_{p} = av^{b}$$

where *a* and *b* are variables, and a plot of logarithmic *i* versus logarithmic *v* should result in a straight line with a slope equal to *b* ranging from 0.5 to 1 (Fig. 2f). A value of *b* = 1 suggests that the current response is directly proportional to the scan rate, indicative of surface capacitive storage stemming from non-Faradaic physical adsorption within the electrical double layer (EDL). Conversely, a value of *b* = 0.5 suggests that the current response is proportional to the square root of the scan rate, characteristic of diffusion-limited Faradaic intercalation. As depicted in Fig. 2f, during bending, the *b*-value was approximately 0.9207, reflecting that the surface capacitive storage is the primary electrochemical process. Meanwhile, during releasing, the *b*-value was approximately 0.7410, demonstrating that diffusion-limited Faradaic intercalation predominates electrochemically. These distinct scenarios, characterized by the value of *b*, offer valuable insights into the electrochemical behavior during bending and releasing, shedding light on the respective roles of surface capacitive storage and diffusion-limited Faradaic intercalation in the system.

### Pseudocapacitive Strain Sensor

**Fig. 3:**
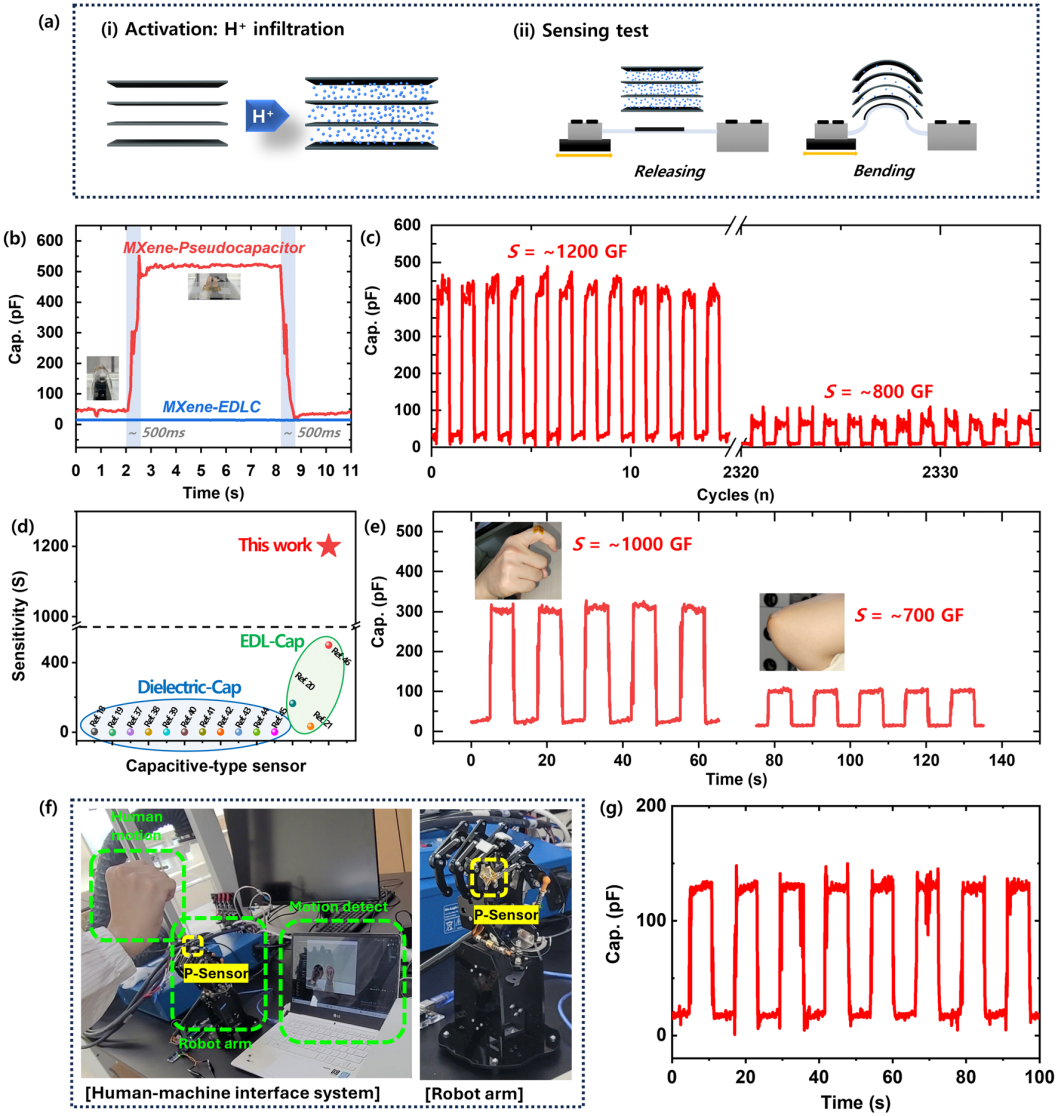
Practical pseudocapacitive sensing test. **a** Schematic illustration of the Pseudocapacitive Sensor; (i) activation: H^+^ ion infiltrating between MXene nanosheets and (ii) sensing test during repetitive bending and releasing (bending: low H^+^ distribution, releasing: high H^+^ distribution). **b** Time response with bending and releasing the sensor at different capacitors of MXene-pseudocapacitor and MXene-EDLC. **c** Pseudocapacitive sensing test of cyclic durability for 2334 bending/releasing cycles at about 1% bending strain. **d** Demonstration of motion detection with the MXene Pseudocapacitive Sensor. For a wearable application, the Pseudocapacitive Sensor is mounted onto the joint of an index finger and attached to the joint of an elbow. **e** Comparison of the sensitivity (*S*) of Pseudocapacitive Sensor in our work with that of previous works on dielectric-cap and EDL-cap sensors. **f** Pseudocapacitive Sensor integrated into a demo prototype for robotics applications and their **g** sensor data

Motion sensing has been extensively studied using strain sensors for human motion detection, soft robotics, and healthcare as human–machine interfaces [18, 34–36]. When considering the practical application of strain sensors, capacitive-type strain sensors are promising candidates due to their excellent linearity (sensitivity to strain) and remarkable responsivity, characterized by low hysteresis in response and recovery [37–40]. However, they face a significant limitation of low sensitivity with the theoretical maximum GF being inherently 1 [33–36]. Here, we apply the unique sensing phenomena observed in MXene flexible interdigitated MSCs during bending and releasing at an extremely small strain to a practical capacitive-type strain sensor (Fig. 3).

Flexible MXene MSCs were fabricated as follow the process outlined in Fig. S1. After obtaining flexible MXene MSCs, to activate the effect of pseudocapacitive sensing, the process of H^+^ ion infiltration between MXene nanosheets was carried out (Fig. 3a–i). As illustrated in Fig. S5, the activation process is as follows. Flexible MXene MSC undergoes charging and discharging cycles 5 times in the bent state, followed by repeating the same steps at the flat state. The infiltration of H^+^ ions was confirmed using ex-situ XRD and ex-situ XPS (Figs. S6 and S7). In the ex-situ XRD data, compared with pure MXene, the 2θ at the (002) peak of ex-situ MXene decreases from 7.12° to 6.52°, where the interlayer spacing increases from 12.40 to 13.54 Å. This indicates that the intercalation of hydronium ions between MXene nanosheets enlarged the interlayer spacing [11, 31, 40]. Meanwhile, in the ex-situ XPS results, as shown in Fig. S7, noticeable peak changes related to -OH terminations were observed at 523.3 eV in the O 1*s* spectra compared with the O 1*s* spectra of pure MXene (Figs. 1g and S7c), whereas Ti 2*p* spectra and C 1*s* spectra exhibited no significant differences before and after charging/discharging [14, 17]. Pseudocapacitve strain sensor testing was performed by repetitive bending and releasing strain (Figs. 3a–(ii) and S5). As indicated in Fig. 3a-(ii), the principle of pseudocapacitive sensing relies on a lower distribution of protons during bending and a higher distribution of protons during releasing. The key to pseudocapacitive sensing lies in the manipulation of proton distribution, which shifts from Faradaic diffusion intercalation to non-Faradaic surface adsorption by altering the *c*-axis alignment of MXene nanosheets. We conducted a detailed study and expanded our experiments to further investigate this phenomenon.

As shown in Fig. 3b–d, we conducted a practical mechanical strain sensing test involving repetitive bending (bending angle: 90°) and releasing, with each cycle lasting 10 s. Figure 3b illustrates the dependence on the type of electrolyte. The MXene-pseudocapacitor (Pseudocapacitive Sensor) was used with an acidic PVA/H_2_SO_4_ electrolyte, while the MXene-EDLC was used with a neutral PVA/LiCl electrolyte (Fig. S8). When subjected to bending, the capacitance of the MXene-pseudocapacitor was 45 pF, while during the release phase, the capacitance increased to 520 pF. The time response and recovery time was approximately 500 ms, and there was no hysteresis.

Figure 3c displays the results of a long-term durability test conducted over 2000 cycles. The strain sensitivity of the Pseudocapacitive Sensor was evaluated by calculating the GF [20, 21]. We expressed the capacitive reactance (*X*_*C*_), which signifies the opposition of a capacitance to changes in current or voltage. *X*_*C*_ is inversely proportional to the signal frequency f (1 kHz) and C (*X*_*C*_ = 1/(2π∙*f*∙*C*)); therefore, the sensitivity (*S*) can be defined as follows [38, 39]:

4 $$S = \left( {\Delta X_{C} /X_{C0} } \right)$$

5 $$\Delta X_{C} = X_{C} - X_{C0}$$

where ε represents the applied strain and *X*_*C*_ and *X*_*C0*_ are the capacitive reactance with and without applied strain, respectively. The sensitivity (*S*) initially reaches an impressive ~ 1200 and maintains a solid ~ 800 even after 2330 cycles.

The *S* value of 1200 achieved in this study surpasses the existing capacitive-type strain sensors, exhibiting an unprecedented sensitivity as demonstrated in Fig. 3d and Table S1. In general, the sensitivity of a dielectric-cap strain sensor theoretically reaches 1. Xu et al. enhanced the sensitivity, achieving a GF of ~ 165 using ionically cross-linked polymer-alginate and covalently cross-linked polymer-polyacrylamide, however, this value remains insufficient for practical applications. The developed Pseudocapacitive Sensor demonstrated nearly perfect linearity and superior sensitivity with a GF of 1200 even at extremely small strains. Additionally, its sensing performance remained stable over 2000 cycles with a rapid response/recovery time of ~ 500 ms, and it achieves superior sensitivity and excellent responsivity while demonstrating low hysteresis in response and recovery. This characteristic enables the development of practical strain sensors with exceptional sensitivity and responsivity, establishing a favorable trade-off relationship.

Pseudocapacitive Sensor to the finger joints and extended elbow attached to the human body (Fig. 3e). The sensitivity (*S*) reached 1000 when applied to the finger joints, and 700 for the extended elbow. Figure S9 demonstrates that there is no change in resistance during bending and releasing. Notably, the sensing attributes remain unaffected by changes in the resistance of the MXene nanosheet, emphasizing their sole dependency on variations in the capacitance of the MXene nanosheet with pseudocapacitive behavior.

Furthermore, we have integrated our Pseudocapacitive Sensor into a robotic prototype (as shown in Fig. 5f, g). The Pseudocapacitive Sensor demonstrated high linearity and sensitivity under extremely small strains, making it particularly suitable for applications involving small-scale strain detection. These findings highlight the strong potential for real-world applications.

### Exploring Pseudocapacitive Sensing Phenomena via Controlling Local pH

**Fig. 4:**
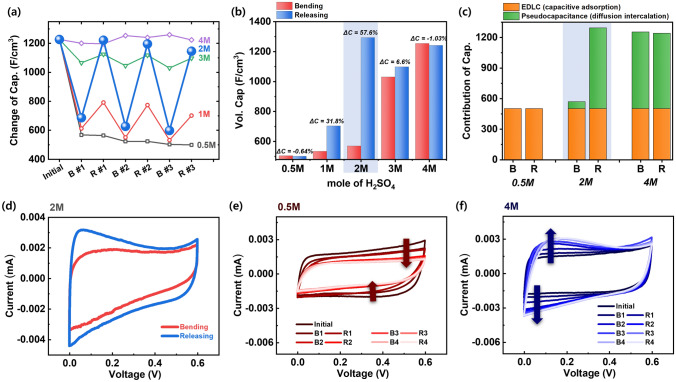
Extension experiment of Pseudocapacitive sensing by controlling of H^+^ molar concentration. Depending on the molarity of the H_2_SO_4_/PVA gel electrolyte of 0.5 – 4 M, **a** variation in capacitances and **b** relative change in capacitance during bending and releasing, showing a pseudocapacitive sensing effective ranging from 1 to 3 M and maximizing sensing effect at 2 M. However, 0.5 and 4 M pseudocapacitive sensing is ineffective. **c** Contribution of capacitance of EDLC (capacitive adsorption) and pseudocapacitor (diffusion-intercalation) at different molarities of 0.5, 2, and 4 M H_2_SO_4_/PVA. CV curves during bending and releasing at **d** 2 M, **e** 0.5 M, and **f** 4 M H_2_SO_4_/PVA at a scan rate of 10 mV s^−1^

We investigated the pseudocapacitive sensing phenomena under diverse conditions by varying the concentration of H^+^ ions in PVA/H_2_SO_4_ electrolyte (Fig. 4). Unlike the EDLC mechanism, the pseudocapacitive charge storage (PC) mechanism stores electric charge through electrochemical redox reactions on the MXene surface [14–17]. Therefore, the portion of the PC mechanism in capacitance would be affected by the concentration of reactant (*i.e.*, pH). The variation in capacitance of MXene-pseudocapacitors with different molar concentrations of H_2_SO_4_ (ranging from 0.5 to 4 M) is depicted in Fig. 4a, b. Pseudocapacitive sensing proves effective within the range of 1 to 3 M H_2_SO_4_ concentrations. Particularly noteworthy is the observation at a 2 M concentration, where the capacitance is 568.17 F cm^−3^ upon bending, while upon releasing, it reaches 1293.27 F cm^−3^. The change in capacitance (ΔC) is maximized at 57.6% in the 2 M H_2_SO_4_ concentration. At the 1 M concentration, when bending, the capacitance is 532.16 F cm^−3^ whereas upon releasing, the capacitance is 701.55 F ^−3^ (Fig. S10); the change in capacitance (ΔC) is 31.8%. At the 3 M concentration, the capacitance is 1029.75 F cm^−3^ upon bending, while upon releasing, it reaches 1097.75 F cm^−3^, and the change in capacitance (ΔC) is 6.6% (Fig. S10).

In contrast, negligible variations in capacitance were observed at concentrations of 0.5 and 4 M. Specifically, at the 0.5 M concentration, the capacitance measures 502.64 F cm^−3^ upon bending and 499.42 F cm^−3^ upon releasing. This corresponds to a slight decrease of -0.6%, with the capacitance stabilizing at a specific low value. Conversely, at the 4 M concentration, the capacitance values are notably higher, registering at 1253.42 F cm^−3^ upon bending and 1240.51 F cm^−3^ upon releasing, resulting in a decrease of only -1.03%. Here, the capacitance converges to a specific high value. Despite these concentration variations, the pseudocapacitive sensing phenomena exhibit reduced effectiveness in both low-molar (0.5 M) and high-molar (4 M) scenarios. Nonetheless, distinct trends emerge at 0.5 and 4 M in the charge storage kinetics, with implications for the dominance of either the EDLC or PC mechanism.

To provide more detailed insights into the charge kinetics on the different molar of H_2_SO_4_ electrolyte (0.5, 2, and 4 M) during bending and releasing, we analyzed the specific contribution to capacitance, specifically in terms of the EDLC or PC mechanism (Fig. 4c). The contribution of capacitance attributed to the EDLC mechanism remained relatively constant at around ~ 500 F cm^−3^ regardless of the H_2_SO_4_ molar concentration. The observed variations stemmed primarily from changes in the PC mechanism portion. At 0.5 M H_2_SO_4_ (higher local pH), the insufficient concentration of H_3_O^+^ near the active sites of MXene precluded the activation of the PC mechanism, resulting in the sole contribution from the EDLC mechanism (Figs. 4c and S10). However, at pH levels above a certain threshold (1 to 4 M H_2_SO_4_), the presence of adequate H_3_O^+^ concentrations facilitated electrochemical redox reactions at the accessible active sites of MXene, thereby enabling the PC mechanism and subsequently increasing its contribution to capacitance (Figs. 4c and S10). These findings suggest that the variations in capacitance within the MXene-hydrogel system reflect changes in the local pH (*i.e.*, local concentration of H_3_O^+^ near accessible active sites of MXene), thus serving as an effective sensing mechanism [47–49].

Upon thoroughly investigating the 2 M case, a significant variation in the contribution of capacitance was observed due to the on/off change of the PC mechanism when bending and releasing. The results indicate that mechanical properties of MXene-hydrogel system can be adjusted by external stresses. When external stress is applied, the degree of anisotropic alignment (DAA) of 2D MXene nanosheets increases, which influences the physical properties [49]. This phenomenon arises from variations in proton distribution resulting from changes in local pH induced by alterations in the DAA. Because of the transition in the charge kinetics between the EDLC mechanism and the PC mechanism, pseudocapacitive sensing phenomena occurred, as evidenced by the CV curves in Fig. 4d.

Meanwhile, at the 0.5 M concentration, only the reaction by the EDLC mechanism exists due to the insufficient participation of H_3_O^+^ ions in the PC mechanism reaction. As seen in Fig. 4e, during the H^+^ activating process with repetitive bending and releasing, the rectangular CV area gradually decreases and eventually saturates. At the 4 M concentration, an excess of H^+^ ions is present, inhibiting any sensing effect from occurring. This surplus of H_3_O^+^ ions fails to induce differences in protonation distribution. As illustrated in Fig. 4f, during the H^+^ activation process with repetitive bending and releasing, the rectangular CV area gradually increases at the redox peaks of ~ 0.15 V (oxidation peak) and ~ 0.05 V (reduction peak) before eventually saturating. Moreover, at the 4 M concentration, a significantly high local hydrogen concentration is generated. Consequently, even under bending conditions, the capacitance remains unchanged, leading to the disappearance of pseudocapacitive sensing characteristics. Despite the vanishing of these characteristics, this can lead to the emergence of another type of component: a highly stable microsupercapacitor exhibiting exceptional cycle stability, with capacitance remaining unaffected even under significant bending. This component holds promise for innovation, particularly in applications such as implantable biomedical devices and health monitoring systems, where flexibility is imperative alongside reliable energy storage properties.

### Degree of Anisotropic Alignment (DAA) of MXene Under Shear Stress

**Fig. 5:**
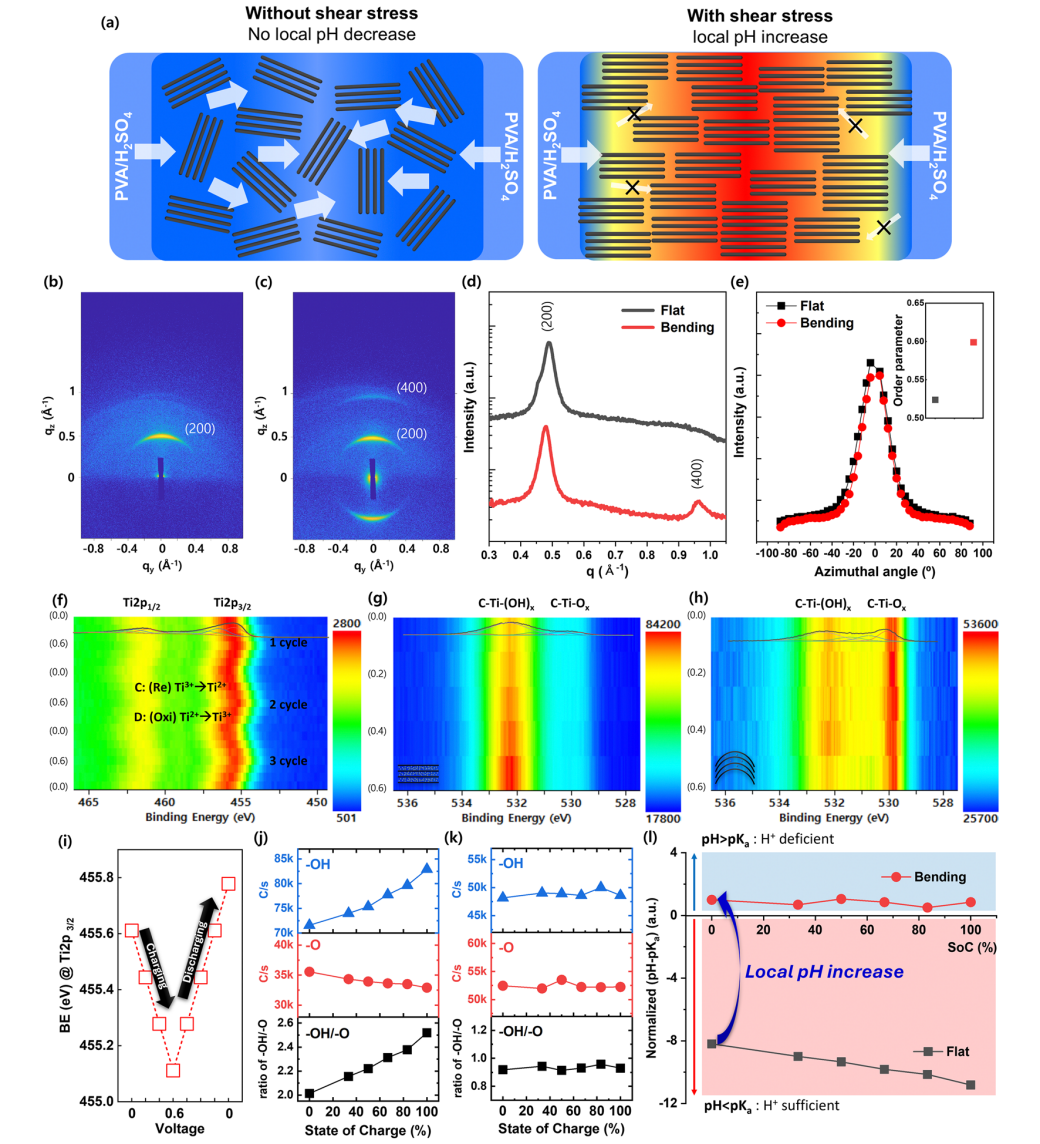
Mechanism study of pseudocapacitive sensing. **a** Changing in the degree of anisotropic alignment (DAA) of MXene nanosheets at bending (with shear stress) and flat (without shear stress), and inducing different local pH tendencies (Flat: no local pH change, Bending: local pH increase). 2D GIWAXS pattern of ex-situ MXene/PVA film in **b** flat and **c** bending geometry and their **d** out-of-plane line cut profile of GIWAXS and **e** Azimuthal profiles of GIWAXS near (200) peak (Inset: calculated order parameter from GIWAXS pattern). **f** In operando XPS Ti 2*p* data of MXene-pseudocapacitor at flat for 3 cycles (C/s: red (2800) to blue (501)), showing peak shifting of Ti 2*p*_3/2_ and Ti 2*p*_1/2_ due to Ti oxidation number change by the electrochemically reduced and oxidized reaction during charging and discharging. **g** In operando XPS O 1*s* data of MXene-pseudocapacitor at flat during charging (C/s: red (84,200 to blue (17,800)) with an increasing peak intensity related to –OH functional group, demonstrating protonation (from –O to –OH). **h** In operando XPS O 1*s* data of MXene-pseudocapacitor at bending during charging (C/s: red (53,600) to blue (25,700)) without the changing of any peak intensity related to the –O or –OH functional group. **i** Detailed 1 cycle of charge–discharge operando XPS Ti 2*p* data of MXene-pseudocapacitor, showing a peak of Ti 2*p*_3/2_ low shift for charging and high shift for discharging. Peak intensities related to the –OH functional group and –O functional group of MXene-pseudocapacitor in accordance with state of charge (SoC) and the ratio of peak intensity of –OH/–O **j** at flat and **k** bending. **l** Normalized (pH-pK_a_) values depending on flat and bending state at different SoC

We conducted a comprehensive investigation into the principles underlying pseudocapacitive sensing phenomena (Fig. 5). As displayed in Fig. 5a, in the absence of applied shear stress (flat configuration), there is no driving force to align the stacked MXene layers. Consequently, the DAA between stacked MXene layers remains low [50], contributing to the lower diffusion barrier for H_3_O^+^ to the accessible active site of MXene by supplying a wide and interconnected diffusion path between stacked MXene. As a result, the sufficient H_3_O^+^ (no local pH increase) can induce both the PC and EDLC mechanism, leading to a high capacitance value (Fig. 5a). When shear stress is applied (bending), it forces alignment of the stacked MXene layers, thereby increasing the DAA between them. This high DAA creates a higher diffusion barrier for H_3_O^+^ to reach the active site, as it supplies separated and narrower diffusion paths between the stacked MXene layers.

The consumption of H_3_O^+^ near the accessible active site of MXene would normally occur at the beginning of the redox reaction; however, the high diffusion barrier disturbs the supply of H_3_O^+^ from the bulk hydrogel. Therefore, the local concentration of H_3_O^+^ decreases (local pH increases) and the PC mechanism cannot occur due to the insufficient reactant near accessible active sites. As a result, the PC mechanism does not contribute to the capacitance, resulting in a lower capacitance value primarily influenced by the EDLC mechanism. In summary, the shear stress adjusts the diffusion barrier for H_3_O^+^ to reach the active sites by changing the DAA, thus modulating the dominance of pseudocapacitive and EDLC mechanisms in charge storage. Previously, studies have reported on the changing alignment of 2D nanosheets due to electrostatic repulsion. For instance, M. Liu et al*.* investigated the anisotropic mechanical properties resulting from electrostatic repulsion between negatively charged unilamellar titanate nanosheets (TiNS) [51]. Additionally, emerging research has focused on the alignment changes of liquid crystalline MXene under mechanical shearing force.

The ordering alignment of MXene was analyzed by ex-situ GIWAXS (Fig. 5b-e). The GIWAXS analysis setup for ex-situ MXene/PVA in the flat and bending states is shown in Fig. S11. The 2D GIWAXS patterns of the ex-situ MXene/PVA in the flat and bending states are exhibited in Fig. 5b, c, and S12, S13, with their corresponding 1D out-of-plane GIWAXS profiles displayed in Fig. 5d. The value of the scattering vector *q* of the (200) peak is 0.489 and 0.478 Å of the ex-situ MXene/PVA film in the flat and bending state, respectively, from which the *d*-spacing is calculated to be approximately 1.28 and 1.31 nm.

Comparing the ordering of ex-situ MXene/PVA between the flat and bending states reveals notable differences. Initially, only a single (200) reflection was observed in the flat configuration, but upon bending, the (400) peak, which was previously not prominently visible, becomes more pronounced, while the intensity of the (200) peak increases. (Fig. 5b–d). These observations suggest that bending under shear force induces anisotropic changes in the alignment ordering of the liquid-crystalline MXenes, as evidenced by the presence of the (400) reflection in the GIWAXS results.

Furthermore, we conducted a comparison of azimuthal (*ϕ*) scans along the (200) peak (Fig. 5e) between the MXene/PVA film in the flat and bending configuration. Notably, the MXene/PVA film while bending exhibited a narrower width than its flat counterpart, indicating higher ordering in the bending film. To quantitatively assess the in-plane ordering, we calculated the orientation order parameter using the following equation [52]:

6 $$S = \frac{{3\cos^{2} \phi - 1}}{2}$$

where

7 $$\cos^{2} \phi = \frac{{\mathop \sum \nolimits_{\phi = 0^\circ }^{90^\circ } I\left( \phi \right)\sin \phi \cos^{2} \phi }}{{\mathop \sum \nolimits_{\phi = 0^\circ }^{90^\circ } I\left( \phi \right)\sin \phi }}$$

Here, *I*(*ϕ*) represents the intensity of the (200) peak at the azimuthal angle *ϕ*. The inset of Fig. 5e illustrates that the MXene/PVA film in a flat configuration exhibited a limited order parameter of 0.524, whereas the bending film demonstrated an improved order parameter of 0.599.

When subjected to bending with a shear strain, an increase in the alignment of MXene flakes occurred, accompanied by a decrease in the accessibility of proton active sites. This phenomenon arises due to the higher diffusion barrier and inadequate presence of H_3_O^+^, wherein the EDLC mechanism predominates. Conversely, upon release to a flat state, the randomness of the MXene flakes is recovered, coupled with the enhanced accessibility of proton active sites. Consequently, this facilitates the kinetics of pseudocapacitive reactions due to the lower diffusion barrier and sufficient presence of H_3_O^+^ ions. The fundamental principle underlying pseudocapacitive sensing involves the manipulation of charge storage kinetics, particularly the transition between the EDLC mechanism (capacitive adsorption) and the PC mechanism (diffusion-intercalation). This transition is achieved by controlling the distribution of accessible H_3_O^+^ ions through alterations in the distribution of MXene flakes in both the flat and bending states.

### In Operando Analyses for an In-Depth Study of Pseudocapacitive Sensing

The pseudocapacitive electrochemical reaction of Ti_3_C_2_T_x_ MXenes is represented as follows [14]:

8 $$Ti_{3} C_{2} O_{x} \left( {OH} \right)_{y} F_{z} + \delta_{e} + \delta H^{ + } \to Ti_{3} C_{2} O_{x - \delta } \left( {OH} \right)_{y + \delta } F_{z}$$

To demonstrate the pseudocapacitive behavior associated with surface redox and protonation tendencies during bending and releasing, we observed changes in the titanium oxidation state and the -O/-OH termination groups using in operando for Ti 2*p* and O 1*s*, respectively (Figs. 5f–l and S14). The in operando XPS environment was established using XPS equipment connected with a potentiostat/galvanostat/impedance analyzer (as detailed in the Method section and Fig. S14).

In operando XPS for Ti 2*p* (Fig. 5f, i, and j), the x-axis represents binding energy (eV), the y-axis indicates the charging state, and color mapping denotes intensities (red: high, blue: low). During charging (oxidation), the oxidation state of Ti increases, evidenced by a decrease in binding energy at Ti 2*p*_3/2_ from 455.6 to 455.1 eV. During discharging (reduction), the oxidation state of Ti decreases, indicated by an increase in binding energy at Ti 2*p*_3/2_ from 455.1 to 455.6 eV. Additionally, as shown in Fig. 5g, the peak position of Ti 2*p*_3/2_ shifts to the right during charging and to the left during discharging for one cycle. Detailed variations of binding energy at Ti 2*p*_3/2_ are presented in Fig. 5h for three cycles. In operando XPS analysis for O 1*s* (Fig. 5i, j) reveals significant variations in the peaks associated with C-Ti-(OH)_x_ and C-Ti-O_x_ between the flat and bending configurations, reflecting changes in the degree of protonation (-O to -OH). In the flat configuration, the peak intensity related to C-Ti-(OH)_x_ at 532.4 eV gradually increases, leading to an elevated -OH/-O ratio during charging (Fig. 5i, k). This suggests that pseudocapacitive kinetics predominantly occur in the flat state. However, in the bending configuration, the intensity of the C-Ti-(OH)_x_ peak at 532.4 eV remains constant, resulting in a stable -OH/-O ratio during charging (Fig. 5j–l). This indicates that EDLC kinetics predominantly occur during bending.

The simplified pseudocapacitive electrochemical reaction of Ti_3_C_2_T_x_ MXene, focusing on the protonation of the oxygen termination group, is as follows:

9 $$Ti - O^{ - } + H^{ + } \to Ti - OH$$

Using this reaction formula, we can get the modified Henderson-Hasselbalch equation as follows [53]:

10 $$pH = pK_{a} + \log \frac{{\left[ {A^{ - } } \right]}}{{\left[ {HA} \right]}}$$

11 $$pK_{a} = pH - \log \frac{{\left[ {Ti - O} \right]}}{{\left[ {Ti - OH} \right]}} = pH + \log \frac{{\left[ {Ti - OH} \right]}}{{\left[ {Ti - O} \right]}}$$

12 $$pH = pK_{a} - \log \frac{{\left[ {Ti - OH} \right]}}{{\left[ {Ti - O} \right]}}$$

where conjugate base (A-) is Ti–O and the weak acid (HA) is Ti–OH. pK_a_ is the negative logarithm of the acid dissociation constant.

Based on the modified Henderson-Hasselbalch equation, in a pH-dependent environment, the electrochemical mechanism of the MXene supercapacitor with an acidic gel electrolyte (PVA/H_2_SO_4_) changes.

We derived locally distributed protons from indirect yet explicit experimental results, driven by the geometric changes of the electrochemical device. By analyzing protonation through peak tendencies related -O and -OH terminations in in-situ O 1*s* XPS spectra at flat and bending states, we indirectly inferred local pH changes and, subsequently, interpreted H + diffusivity.

By comparing the ‘normalized pH-pK_a_’ values at flat and bending states, calculated using the modified Henderson-Hasselbalch equation and derived from in-situ XPS data, we can anticipate a different local pH trend (i.e., proton distribution) between at flat and bending condition.

As shown in Fig. 5j, under the bending condition, the ratio of [-OH]/[-O] is less than 1 in the in-situ XPS data, and according to the modified Henderson-Hasselbalch equation, the normalized pH-pK_a_ is positive (Fig. 5l). This indicates a proton-deficient environment at the interface, where protons are scarce, leading to a lack of H^+^-related reactions. As a result, only the EDLC mechanism is active. Supporting this, no ΔpH is observed during charging.

In contrast, under the flat state in Fig. 5k, the ratio of [-OH]/[-O] is greater than 1 in the in-situ XPS data, and the normalized pH-pK_a_ is negative according to the modified Henderson-Hasselbalch equation (Fig. 5l). This suggests a proton-rich environment at the interface, where protons are abundant and participate in reactions. Therefore, both the PC and EDLC mechanisms are active. As evidence of this, a ΔpH is observed during charging.

A similar trend was also observed in operando Raman spectroscopy (Fig. S16). The Raman spectra of Ti_3_C_2_T_x_ MXene are influenced by various factors such as different surface terminations, intercalated species, and adsorbed species [27–29]. The region of 230 – 470 cm^−1^ represents in-plane (*E*_g_) vibrations of surface groups attached to titanium atoms, which could potentially be utilized to probe the surface chemistry of MXene. During charging in the flat configuration, active chemisorption reactions take place on the MXene surface, inducing alterations in its surface chemistry. These changes are manifested in the Raman spectra, reflecting the evolving nature of the material during the charging process. Conversely, in the bending configuration, physisorption predominantly occurs, with minimal involvement of chemisorption related to -OH terminations, resulting in no noticeable change in peak shape.

## Conclusions

In this study, we developed a pioneering device named the Pseudocapacitive Sensor, which demonstrates a unique sensing mechanism based on changes in local pH and proton distribution during bending (shearing). This pseudocapacitive sensing attribute is highlighted by the significant variation in capacitance as the MXene-based supercapacitors undergo bending and releasing. This phenomenon results from the different proton distributions caused by shearing, which leads to changes in local pH and subsequently alters the energy storage kinetics.

In practical terms, the Pseudocapacitive Sensor exhibits an ultra-high sensitivity of approximately 1200 S GF under extremely small strain, with enhanced response properties. This high sensitivity is attributed to the shift in energy storage kinetics from pseudocapacitance (involving protonation) to electrical double layer capacitance (EDLC) during bending.

Furthermore, the Pseudocapacitive Sensor demonstrated exceptional cycle stability, maintaining its high sensitivity and capacitance performance after more than 2000 bending and releasing cycles. This emphasizes the robustness of the device for long-term practical applications.

Our in-operando analysis further confirmed the changes in the titanium oxidation state and protonation of oxygen functional groups during bending, providing detailed insights into the underlying electrochemical mechanisms.

The optimal performance of the Pseudocapacitive Sensor was achieved at a 2 M concentration of H_2_SO_4_, where the capacitance change was maximized. Lower and higher concentrations showed reduced effectiveness, indicating the crucial role of proton concentration in achieving optimal sensing performance.

This study introduces a multifunctional MXene-based device capable of serving both as a micro-supercapacitor for energy storage and as a highly sensitive pseudocapacitive strain sensor. This dual functionality expands its potential applications in fields such as advanced robotics, implantable biomedical devices, and health monitoring systems, enabling a range of future technological advancements.

## Supplementary Information

Below is the link to the electronic supplementary material.

Supplementary file1 (DOCX 5873 KB)
