# Supplementary material for: Unlocking Novel Functionality: Pseudocapacitive Sensing in MXene-Based Flexible Supercapacitors
Source: Nanomicro Lett. 2024 Dec 9;17:86. doi: 10.1007/s40820-024-01567-2 (PMC11628472; doi:10.1007/s40820-024-01567-2)
Supplement: Supplementary file 1 — Supplementary file1 (DOCX 5873 KB) [file 40820_2024_1567_MOESM1_ESM.docx]

Supporting Information for

**Unlocking Novel Functionality: Pseudocapacitive Sensing in MXene-Based Flexible Supercapacitors**

Eunji Kim^1,2^, Seongbeen Kim^1^, Hyeong Min Jin^3,4^, Gyungtae Kim^2^, Hwi-Heon Ha^2^, Yunhui Choi^2^, Kyoungha Min^2^, Su-Ho Cho^2^, Hee Han^2^, Chi Won Ahn^2^, Jaewoo Roh^6^, Il-Kwon Oh^6^, Jinwoo Lee^1,^*, Yonghee Lee^2,5^*

^1^Department of Chemical and Biomolecular Engineering, Korea Advanced Institute of Science and Technology (KAIST), 291 Daehak-Ro, Yuseong-Gu, Daejeon, 34141 Republic of Korea

^2^National Nano Fab Center (NNFC), 291 Daehak-Ro, Yuseong-Gu, Daejeon 34141, Republic of Korea

^3^Department of Organic Materials Engineering, Chungnam National University, 99 Daehak-ro, Yuseong-gu, Daejeon 34134, Republic of Korea

^4^Department of Materials Science and Engineering, Chungnam National University, 99 Daehak-ro, Yuseong-gu, Daejeon 34134, Republic of Korea

^5^Department of Nano & Advanced Materials Science and Engineering, Kyungpook National University, 2559, Gyeongsang-daero, Sangju-si, Gyeongsangbuk-do, 37224, Republic of Korea

^6^Department of Mechanical Engineering, Korea Advanced Institute of Science and Technology (KAIST), 291 Daehak-Ro, Yuseong-Gu, Daejeon, 34141 Republic of Korea

*Corresponding authors. E-mail: [jwlee1@kaist.ac.kr](mailto:jwlee1@kaist.ac.kr) (Jinwoo Lee); [yhlee@knu.ac.kr](mailto:yhlee@knu.ac.kr) (Yonghee Lee)

**Supplementary Figures and Tables**


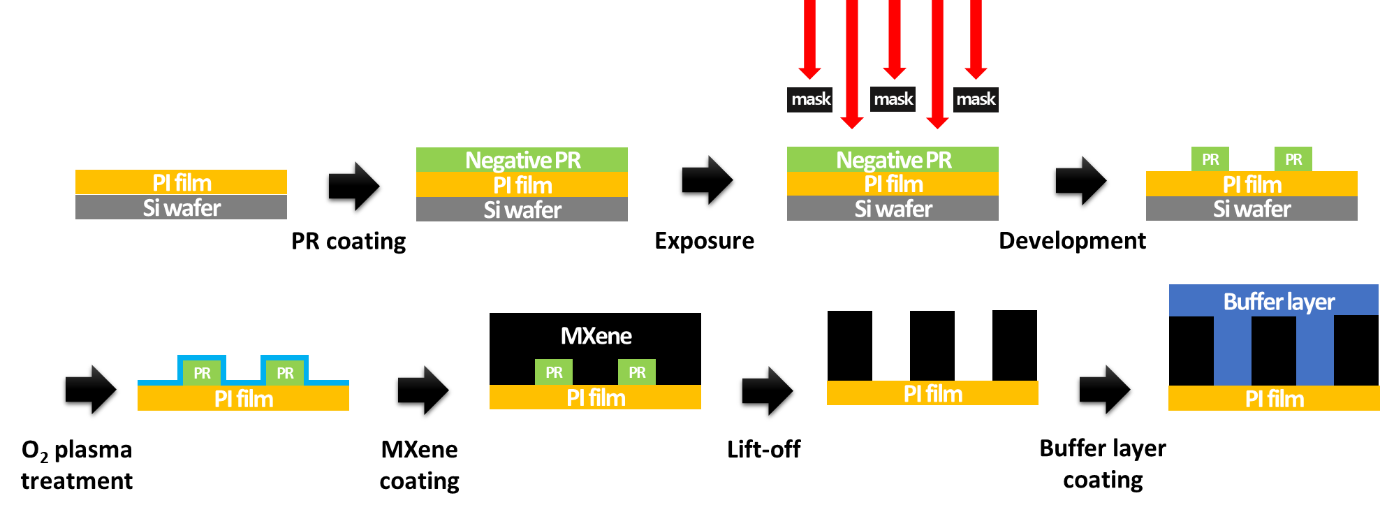


**Fig. S1** Fabrication process of flexible MXene MSCs: 1) photoresist coating (negative PR: L300, width: 50 µm, spacing: 50 µm, length: 3.95 mm, # of finger: 60 ea, thickness: 3.5 µm), 2) exposure (exposure time: 4~15s), 3) development, 4) O_2_ plasma treatment (100W, 20sccm, 1min), 4) MXene coating (10-15 mg ml^-1^ of MXene solution, spin coating: 1000-1500rpm, 5min), 5) lift-off using by acetone (sonication), 6) buffer layer coating (10 wt% PVA in water, spin coating: 1000rpm, 1min)


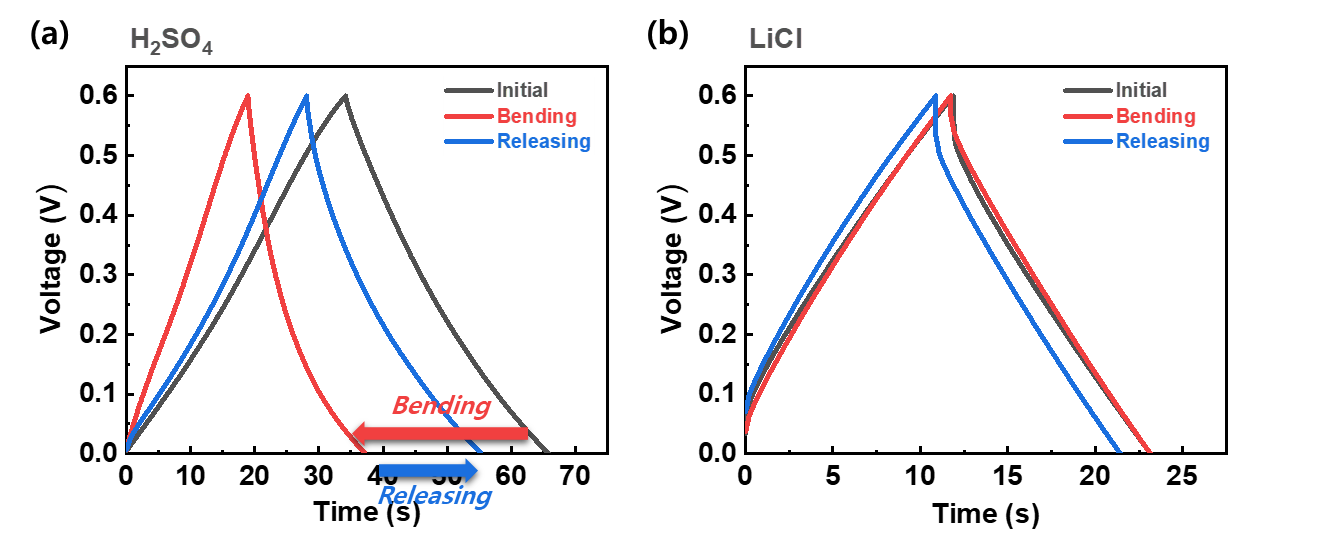


**Fig. S2** Electrochemical sensing test of flexible MXene MSCs by GCD (galvanostatic charge-discharge) on **a**) PVA/H_2_SO_4_ and **b**) PVA/LiCl electrolyte


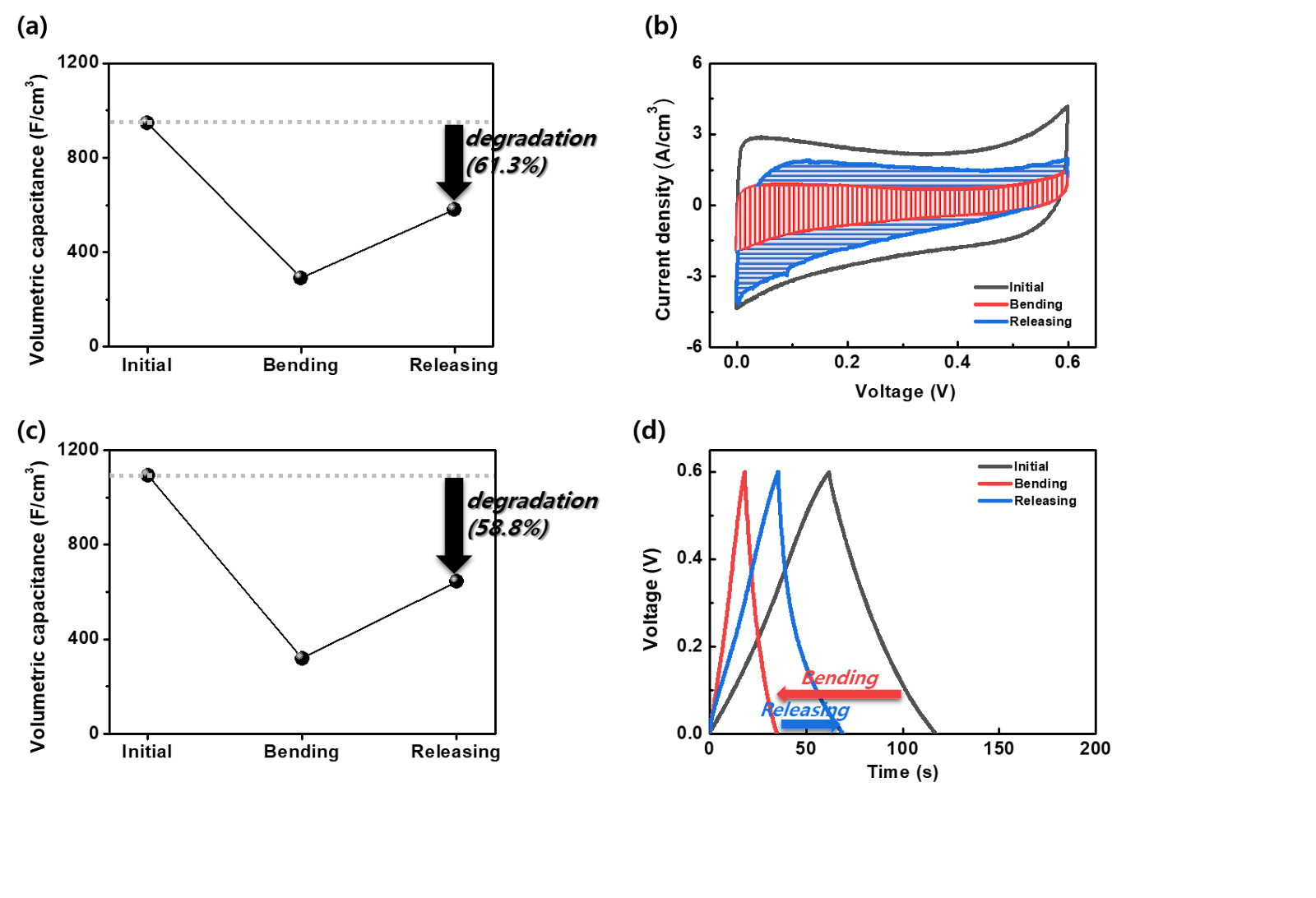


**Fig. S3.** Electrochemical sensing test without buffer layer. **a**) change in capacitance calculated by **b**) CV curves, and **c**) change in capacitance calculated by d) GCD plot


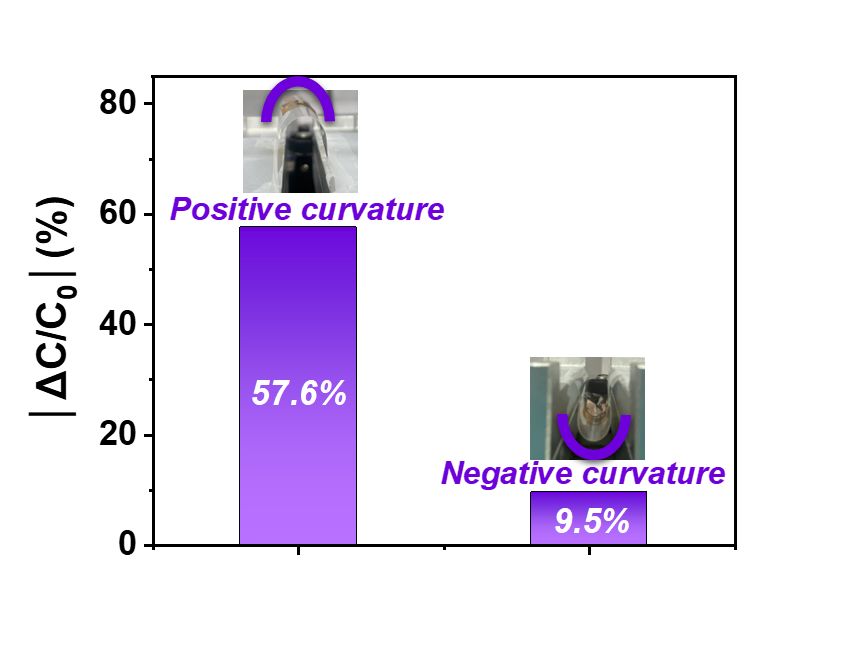


**Fig. S4** Electrochemical sensing test depending on the curvature direction of positive (tensile strain) and negative (compressive strain)


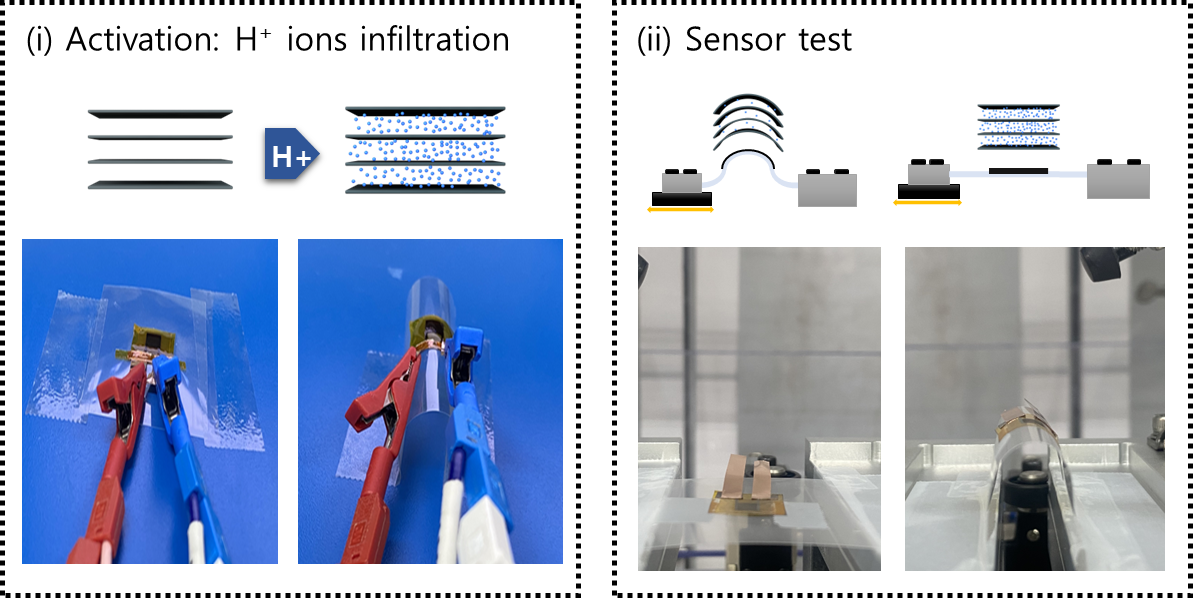


**Fig. S5** Process of (**i**) Activation (H+ ions infiltration) and (**ii**) mechanical sensing test in practical


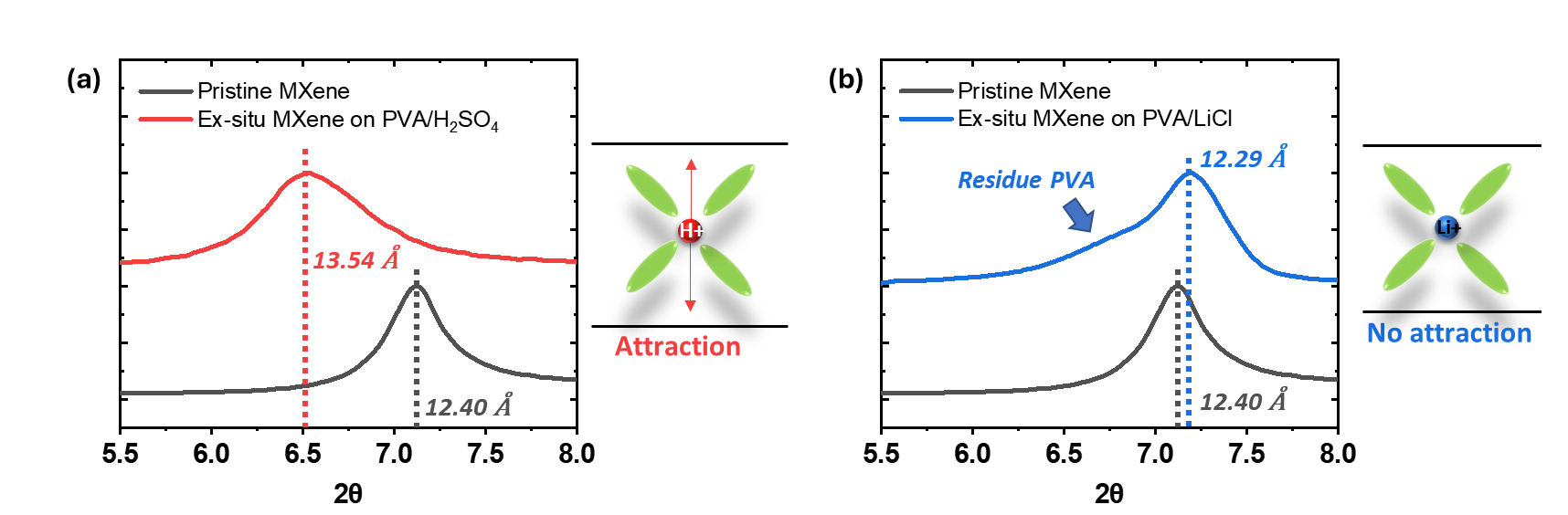


**Fig. S6** Ex-situ XRD of MXene films on **a**) PVA/H_2_SO_4_ electrolyte (*d*-spacing : pristine (12.40 Å) and PVA/H_2_SO_4_ (13.54 Å)) and **b**) PVA/LiCl electrolyte (*d*-spacing : pristine (12.40 Å) and PVA/H_2_SO_4_ (12.29 Å)


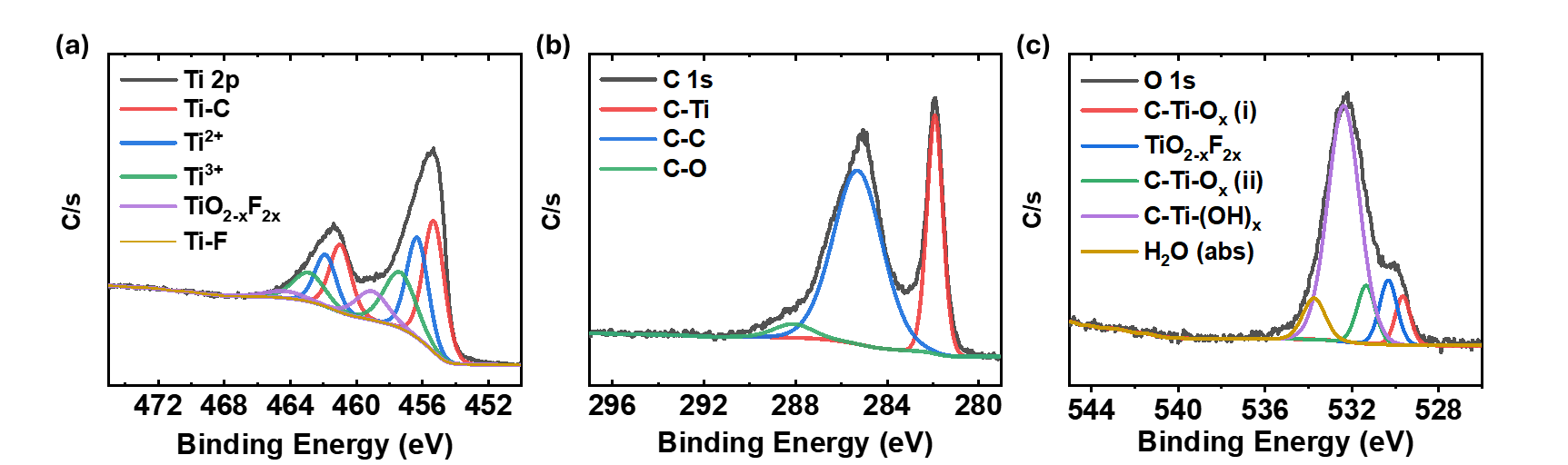


**Fig. S7** Ex-situ XPS spectra of MXene supercapacitors on PVA/H_2_SO_4_ electrolyte after 100 cycles charge/discharge. **a**) Ti 2p, **b**) C 1s, and **c**) O 1s, respectively


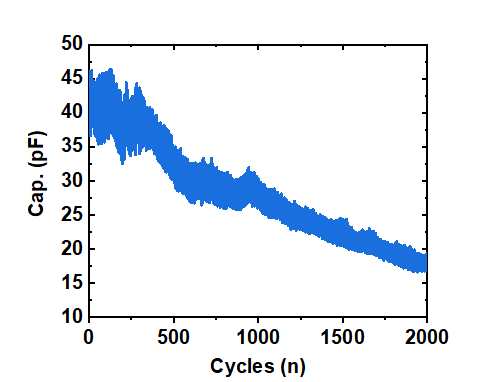


**Fig. S8** Mechanical sensing test of MXene-EDLC


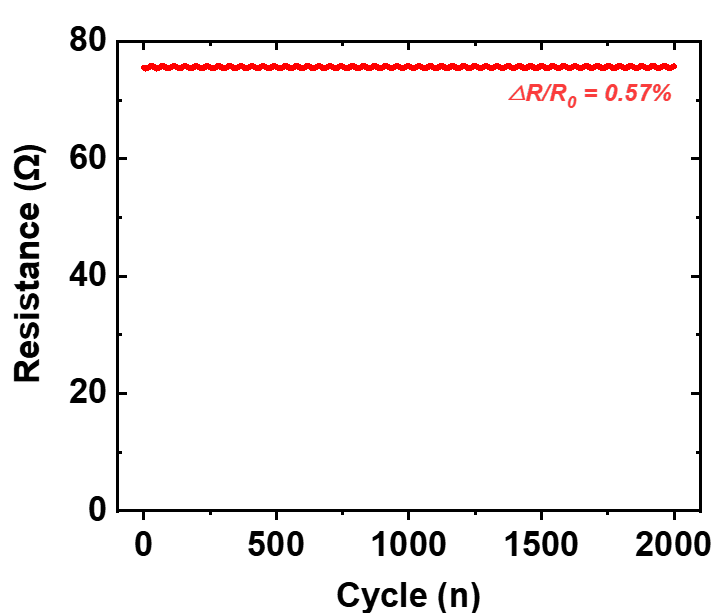


**Fig. S9** The change of resistance of MXene during bending and releasing up to 2,000 cycles

**Table S1** Comparison of capacitive-type strain sensors

|  | **Material** | **Sensor type** | **Linearity** | **Sensitivity** | **Strain (ε) (%)** | **Stability** | **Response time (ms)** | **Refs.** |
| --- | --- | --- | --- | --- | --- | --- | --- | --- |
| **Pseudocapacitor-cap** | **MXene/PVA/H_2_SO_4_** | **Capacitive**  **(interdigitated)** | **Near one-linear** | **GF 1,200** | **~1%** | **up to 2,000** | **~500 ms** | **Our work** |
| Dielectric-cap | Au film/Parylene/Prestretched elastomer | Capacitive  (sandwich) | One-linear | GF 3.05 | 20%  upto 110% | 1,000 | NA | [S1] |
|  | AgNW/PDMS | Capacitive  (interdigitated) | One-linear | GF -2 | upto 30% | 1,000 | NA | [S2] |
|  | CNTs–Dragon-skin elastomer | Capacitive  (sandwich) | One-linear | GF 0.97 | 1%  upto 300% | 2,000 | ~100 ms | [S3] |
|  | CNTs–silicone elastomer | Capacitive  (sandwich+pattern) | One-linear | GF 0.99 | 100% | 3,000 | NA | [S4] |
|  | AgNWs–Ecoflex | Capacitive  (sandwich) | One-linear | GF 0.7 | upto 50% | 100 | ~40 ms | [S5] |
|  | AgNWs-PDMS | Capacitive  (sandwich) | One-linear | GF 1.01 | 40% | NA(4) | NA | [S6] |
|  | EGaIn/Acrylic elastomer/  Polysil/silicone elastomer | Capacitive  (interdigitated) | One-linear | GF 1.61 | 80% | 10,000 | NA | [S7] |
|  | Graphene/PDMS | Capacitive  (sandwich) | One-linear | GF 0.97 | 80% | 1,000 | 180 ms | [S8] |
|  | CNTs/PDMS | Capacitive  (interdigitated) | One-linear | GF 0.637 | 100% | 1,500 | 60 ms | [S9] |
|  | MXene/VHB/PVA hydrogel | Capacitive  (sandwich) | One-linear | GF 0.4 | 200% | 10,000 | 190 ms | [S10] |
|  | Ecoflex-CB | Capacitive  (sandwich) | non-linear | GF 0.83-0.98 | 50-500% | 10,100 | NA | [S11] |
|  | SWCNT-SB/Ecoflex | Capacitive  (sandwich) | non-linear | GF -0.64-1.13 | 0-100% | 10 | 80ms | [S12] |
|  | PA 6.6/elastane yarn | Capacitive  (interdigitated) | One-linear | GF -0.68 | 230% | 100 | 66ms | [S13] |
|  | SSPU/Galinstan | Capacitive  (sandwich) | One-linear | GF 1.003 | 200% | NA | 32ms | [S14] |
|  | Parylene C-SU-8 | Capacitive  (sandwich) | One-linear | GF -0.25 | 100% | 100 | 22ms | [S15] |
|  | Chitosan/Cu NWs | Capacitive  (sandwich) |  | GF 2.9 | 70% | 1,000 | NA | [S16] |
| EDL-cap | Ag NFs/Ionic Hydrogels | Capacitive  (sandwich) | Two-linear | GF 3  GF165 | 10%  1,000% | 300 | 320 ms | [S16] |
|  | PDA–PAA–Cr3+ hydrogel | Capacitive  (sandwich) | Non-linear | GF33.4 | 500% | NA (10) | NA | [S17] |
|  | Poly(NaSS-Co-DMC) | Capacitive  (sandwich) | Two-linear | GF 2.9 | 0-350% | 1,000 | 250ms | [S18] |


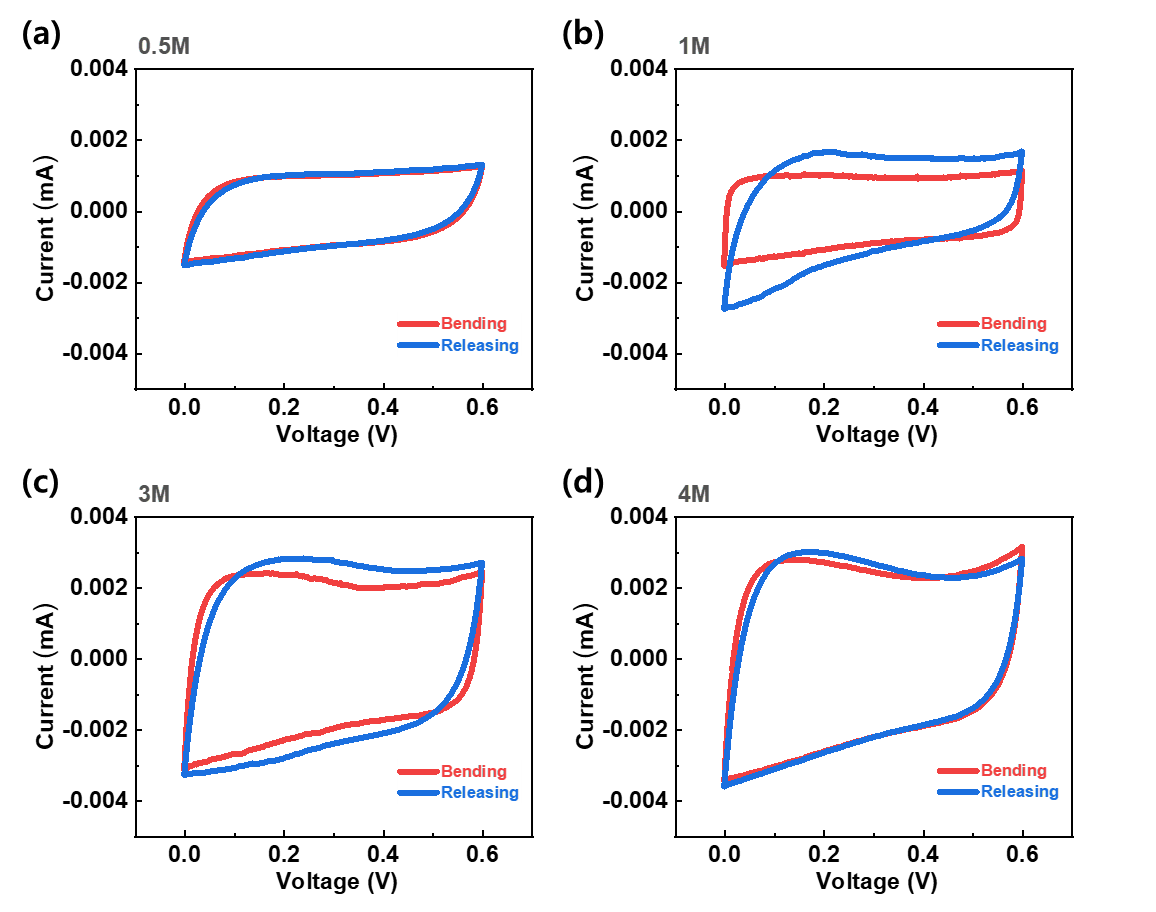


**Fig. S10** **a–d**) CV curves depending on varying molar concentration of PVA/H_2_SO_4_ electrolyte (0.5M – 4M) on bending and releasing


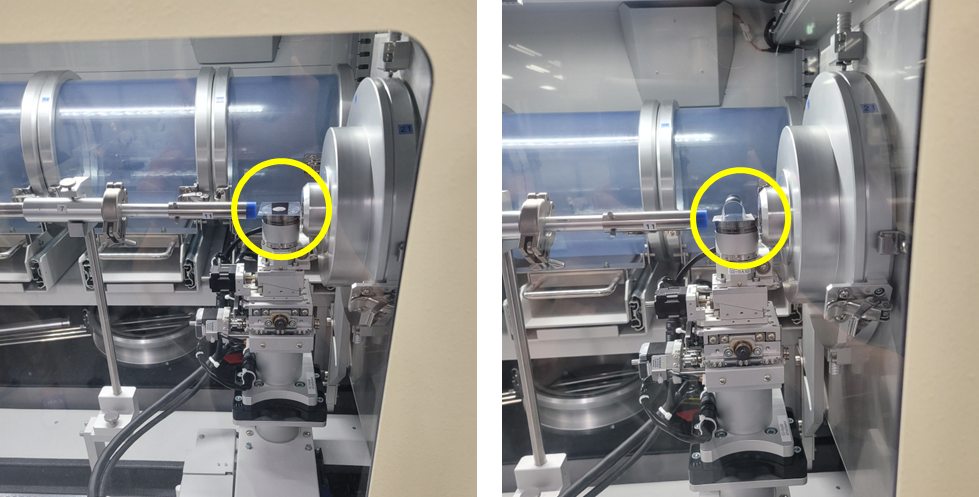


**Fig. S11** GIWAXS analysis set up of MXene films in flat and bent


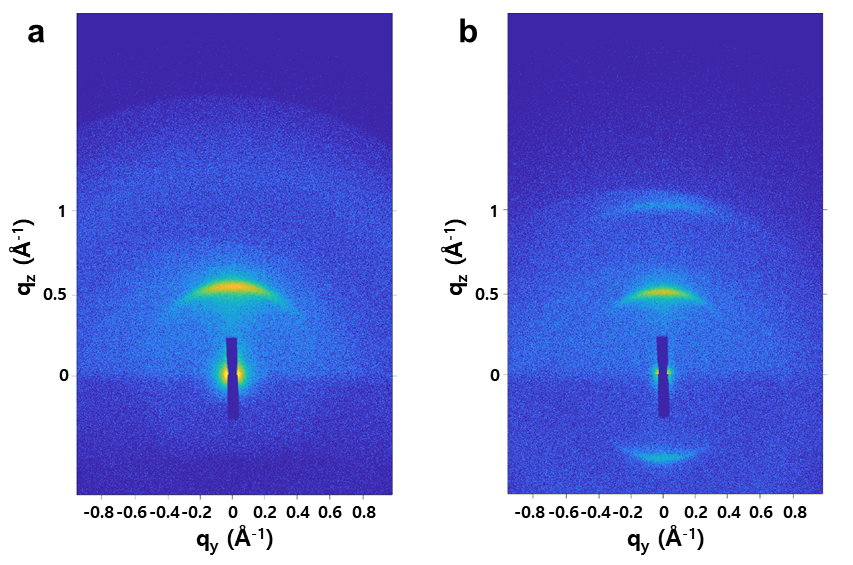


**Fig. S12** 2D GIWAXS pattern of MXene film in **a**) flat and **b**) bending geometry


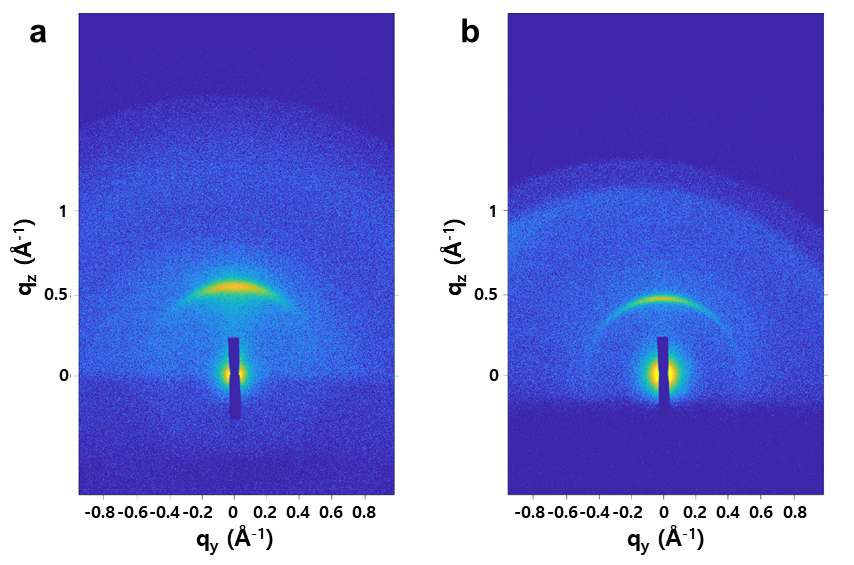


**Fig. S13** 2D GIWAXS pattern of **a**) MXene and **b**) MXene/PVA film under compression strain

**
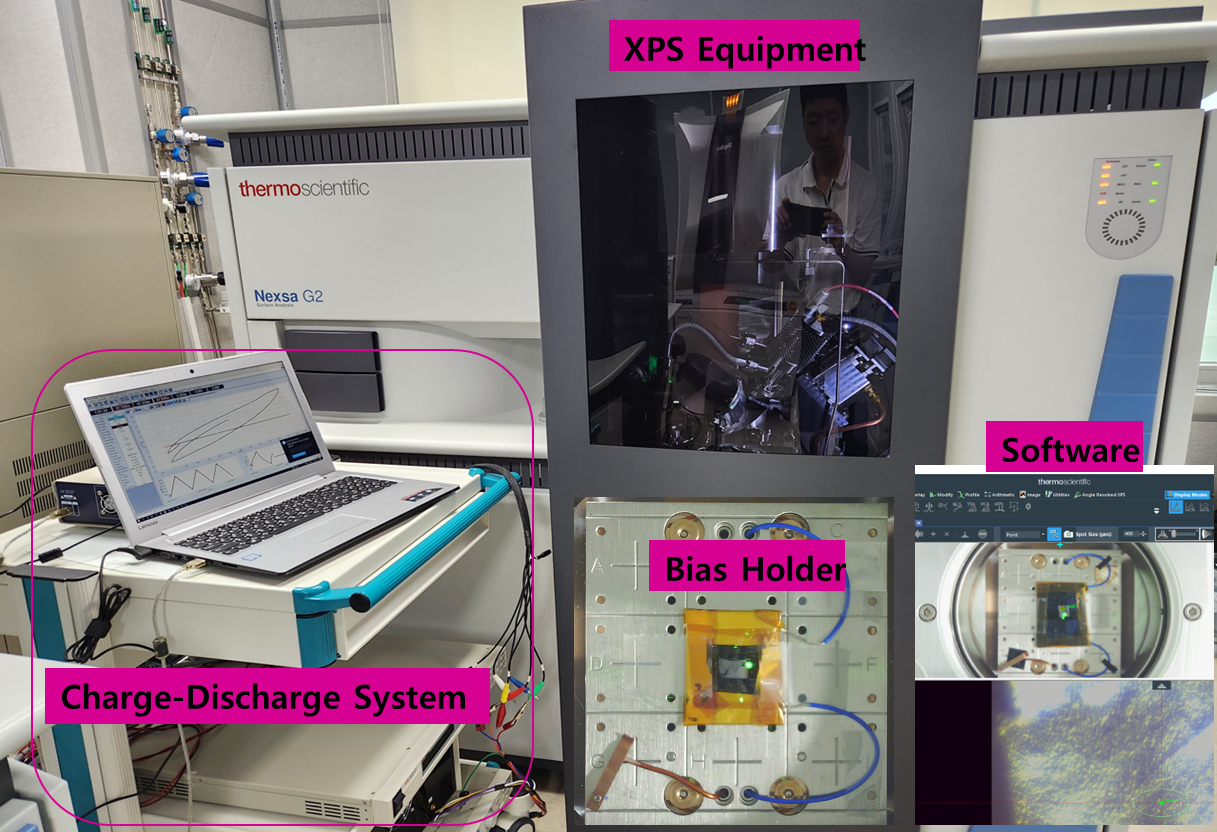
**

**Fig. S14** Operando XPS experiment set-up environment (XPS equipment connected with charge-discharge system)


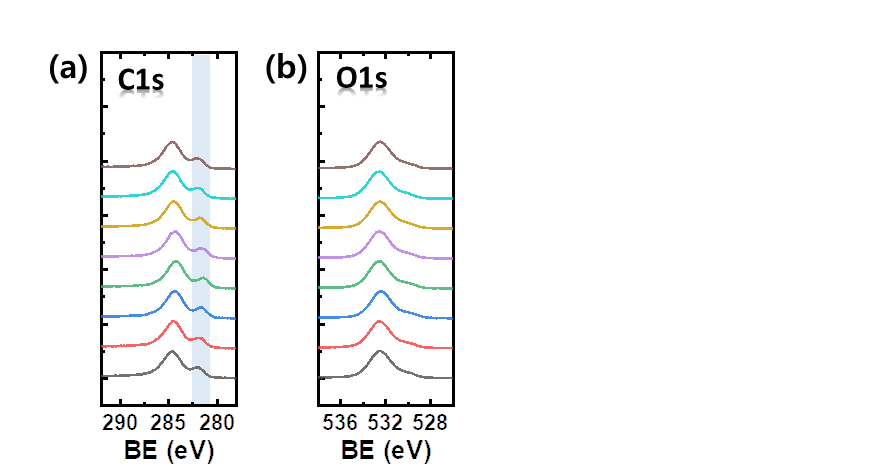


**Fig. S15** Operando XPS spectra at **a**) C 1s and **b**) O 1s of MXene SCs


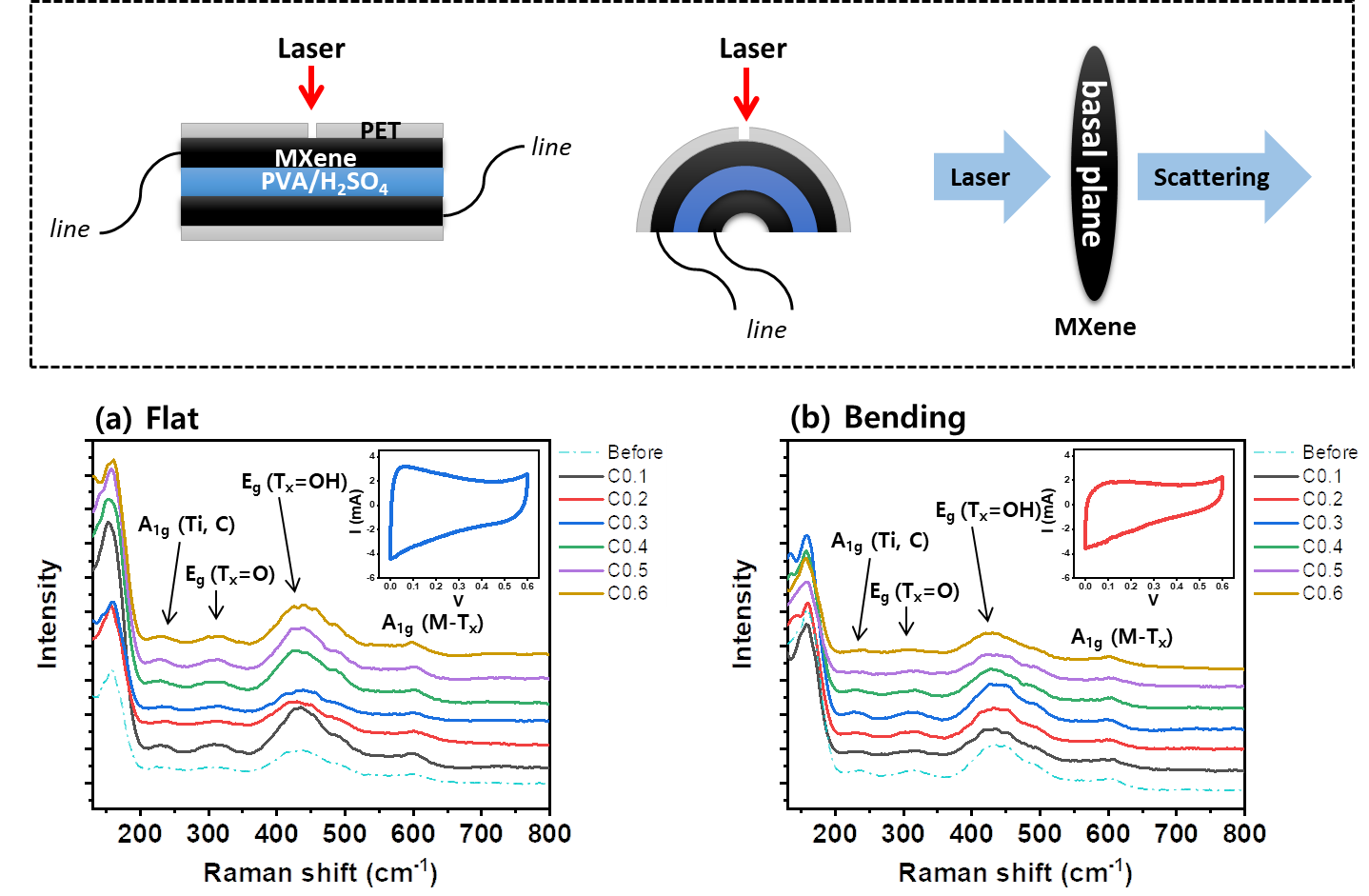


**Fig. S16** Operando Raman spectra of **a**) MXene SC in flat state and **b**) in bent state

**Supplementary References**

1. R. Nur, N. Matsuhisa, Z. Jiang, M. O. G. Nayeem, T. Yokota et al., A highly sensitive capacitive-type strain sensor using wrinkled ultrathin gold films. Nano Lett. **18**(9), 5610-5617 (2018). <https://doi.org/10.1021/acs.nanolett.8b02088>
2. S.-R. Kim, J.-H. Kim, J.-W. Park, Wearable and transparent capacitive strain sensor with high sensitivity based on patterned Ag nanowire networks. ACS Appl. Mater. Inter. **9**(31), 26407-26416 (2017). <https://doi.org/10.1021/acsami.7b06474>
3. L. Cai, L. Song, P. Luan, Q. Zhang, N. Zhang et al., Super-stretchable, transparent carbon nanotube-based capacitive strain sensors for human motion detection. Sci. Rep. **3**(1), 3048 (2013). <https://doi.org/10.1038/srep03048>
4. D. J. Cohen, D. Mitra, K. Peterson, M. M. Maharbiz, A highly elastic, capacitive strain gauge based on percolating nanotube networks. Nano Lett. **12**(4), 1821-1825 (2012). <https://doi.org/10.1021/nl204052z>
5. S. Yao, Y. Zhu, Wearable multifunctional sensors using printed stretchable conductors made of silver nanowires. Nanoscale **6**(4), 2345-2352 (2014). <https://doi.org/10.1039/C3NR05496A>
6. P. Goel, J. P. Singh, Fabrication of silver nanorods embedded in pdms film and its application for strain sensing. J. Phys. D: Appl. Phys. **48**(44), 445303 (2014). <https://doi.org/10.1088/0022-3727/48/44/445303>
7. J. Shintake, T. Nagai, K. Ogishima, Sensitivity improvement of highly stretchable capacitive strain sensors by hierarchical auxetic structures. Front. Robot. AI. **6**((2019).
8. C. Deng, L. Lan, P. He, C. Ding, B. Chen et al., High-performance capacitive strain sensors with highly stretchable vertical graphene electrodes. J. Mater. Chem. C **8**(16), 5541-5546 (2020). <https://doi.org/10.1039/D0TC00491J>
9. X. Wang, Y. Deng, P. Jiang, X. Chen, H. Yu, Low-hysteresis, pressure-insensitive, and transparent capacitive strain sensor for human activity monitoring. Microsyst. Nanoeng. **8**(1), 113 (2022). <https://doi.org/10.1038/s41378-022-00450-7>
10. J. Zhang, L. Wan, Y. Gao, X. Fang, T. Lu et al., Highly stretchable and self-healable mxene/polyvinyl alcohol hydrogel electrode for wearable capacitive electronic skin. Adv. Electron. Mater. **5**(7), 1900285 (2019). https://doi.org/10.1002/aelm.201900285
11. J. Shintake, Y. Piskarev, S. H. Jeong, D. Floreano, Ultrastretchable strain sensors using carbon black-filled elastomer composites and comparison of capacitive versus resistive sensors. Adv. Mater. Technol. **3**(3), 1700284 (2018). <https://doi.org/https://doi.org/10.1002/admt.201700284>
12. K. Okada, T. Horii, Y. Yamaguchi, K. Son, N. Hosoya et al., Ultraconformable capacitive strain sensor utilizing network structure of single-walled carbon nanotubes for wireless body sensing. ACS Appl. Mater. Inter. **16**(8), 10427-10438 (2024). <https://doi.org/10.1021/acsami.3c19320>
13. A. F. Yilmaz, I. A. K. Ahmed, C. Gumus, K. Ozlem, M. S. Cetin et al., Highly stretchable textile knitted interdigital sensor for wearable technology applications. Adv. Sens. Res. **3**(2), 2300121 (2024). https://doi.org/10.1002/adsr.202300121
14. F. Sun, L. Liu, T. Liu, X. Wang, Q. Qi et al., Vascular smooth muscle-inspired architecture enables soft yet tough self-healing materials for durable capacitive strain-sensor. Nat. Comm. **14**(1), 130 (2023). <https://doi.org/10.1038/s41467-023-35810-y>
15. X. Huang, L. Liu, Y. H. Lin, R. Feng, Y. Shen et al., High-stretchability and low-hysteresis strain sensors using origami-inspired 3D mesostructures. Sci. Adv. **9**(34), eadh9799 <https://doi.org/10.1126/sciadv.adh9799>
16. H. Xu, Y. Lv, D. Qiu, Y. Zhou, H. Zeng et al., An ultra-stretchable, highly sensitive and biocompatible capacitive strain sensor from an ionic nanocomposite for on-skin monitoring. Nanoscale **11**(4), 1570-1578 (2019). <https://doi.org/10.1039/C8NR08589G>
17. V. K. Rao, N. Shauloff, X. Sui, H. D. Wagner, R. Jelinek, Polydiacetylene hydrogel self-healing capacitive strain sensor. J. Mater. Chem. C **8**(18), 6034-6041 (2020). <https://doi.org/10.1039/D0TC00576B>
18. H. Zheng, H. Zhou, Z. Wang, S. Zhang, H. Zhang, Ionically conductive and self-healing polyampholyte hydrogels for wearable resistive strain sensors and capacitive pressure sensors. ACS Appl. Polymer Mater. **5**(9), 7581-7589 (2023). <https://doi.org/10.1021/acsapm.3c01497>
